# Identification and Validation of the Key Genes of Diabetic Vasculopathy: Evidence Based on Bioinformatics Analysis and Animal Study

**DOI:** 10.1155/ijog/7850852

**Published:** 2025-10-24

**Authors:** Feng Li, Chi Geng, Xing Xu, Yu-Lun Zhou, Xin-Ru Guo, Rui-Tao Wang, You Zhang, Si-Liang Peng, Meng-Chao Jin, Jian Huang, Hui-Yu Bai, Hui Li, Xiao-Song Gu, Songyun Zhao

**Affiliations:** ^1^ Department of Cardiology, The Second Affiliated Hospital of Soochow University, Suzhou, Jiangsu, China, suda.edu.cn; ^2^ School of Biology & Basic Medical Science, Soochow University, Suzhou, Jiangsu, China, scu.edu.tw

**Keywords:** animal study, bioinformatics analysis, diabetic vasculopathy, key genes, Type 2 diabetes milieus

## Abstract

**Backgrounds:**

The mechanisms contributing to diabetic vasculopathy have not been fully understand.

**Methods:**

First, we identified differentially expressed genes (DEGs) of diabetic vasculopathy via GSE13760. Enrichment analysis was conducted. We utilized the cMAP database to identify potential small‐molecular drugs targeting diabetic vasculopathy based on upregulated DEGs. Hub genes were extracted from protein–protein interaction (PPI) networks, and their expression and correlation patterns were further evaluated. Key genes implicated in diabetic vasculopathy were determined by integrating three distinct algorithmic approaches. Additionally, we constructed mRNA–miRNA and mRNA–transcription factor (TF) regulatory networks and performed immune infiltration as well as single‐cell RNA sequencing (scRNA‐seq) analyses. Finally, animal studies were carried out to provide preliminary experimental validation.

**Results:**

One hundred thirty‐nine DEGs were identified in the comparison between Type 2 diabetes mellitus (T2DM) and control (Con) arterial samples. Then, enrichment analysis revealed that the DEGs were associated with several key pathways, including cytokine–cytokine receptor interaction, regulation of phosphatidylinositol 3‐kinase activity, and the TGF‐beta signaling pathway. Ten leading small‐molecular compounds with therapeutic potential for diabetic vasculopathy were identified. Among the upregulated genes, BMP4 and LEP were selected as key candidates. Regulatory network analyses, including mRNA–miRNA and mRNA–TF interactions, along with immune infiltration profiling, suggested that multiple miRNAs, TFs, and immune cells may collectively influence BMP4 and LEP expression. scRNA‐seq further indicated predominant expression of BMP4 within endothelial cells and fibroblasts. Finally, experimental validation in the T2DM mouse model corroborated the expression patterns of these key genes and enrichment findings.

**Conclusions:**

Findings indicate that the two upregulated genes, BMP4 and LEP, are implicated in the pathophysiology of diabetic vasculopathy.

## 1. Introduction

Diabetes is a common metabolic disease in clinics, which is estimated that approximately 8% of adults in developed countries have Type 2 diabetes mellitus (T2DM) by 2035 [1]. Importantly, diabetic patients have been reported to be at a significantly higher risk (up to 10‐fold) of experiencing cardiovascular events compared with age‐matched nondiabetic individuals, subsequently leading to an increasing medical burden and premature mortality [2].

Diabetic vasculopathy mainly includes macrovascular disease and microvascular disease. Diabetic arterial lesion is a key pathophysiological change in diabetic vasculopathy, including inflammatory activation, increased extracellular matrix, arterial plaque formation, and arterial fibrosis and remodeling, subsequently leading to multiple arterial diseases (such as arterial stiffness, atherosclerosis, aortic aneurysm, and aortic dissection) [3]. Reportedly, several possible mechanisms, such as immune cell activation, inflammation, oxidative stress, advanced glycation end‐products increase, and microRNAs changes, might be responsible for the initiation and progression of diabetic vasculopathy [4], whereas the mechanisms contributing to diabetic vasculopathy have not been fully understood, which leads, to an extent, to unsatisfied therapy effect, irreversible disease progression, and relatively poor prognosis for diabetic vasculopathy.

This study sought to screen key genes, as well as elucidate the underlying mechanisms of diabetic vasculopathy by combining bioinformatics analysis with animal experiments. We first identified differentially expressed genes (DEGs) between T2DM arterial samples and Con samples via GSE13760 gene expression profile. We performed enrichment analyses via multiple methods, including gene set enrichment analysis (GSEA), Metascape, and ClueGo, to explore the mechanisms underlying diabetic vasculopathy. The potential small‐molecular drugs for diabetic vasculopathy treatment via connectivity map (cMAP) online database with upregulated DEGs were screened. We conducted protein–protein interaction (PPI) network and identified the possible hub genes using four algorithms in Cytoscape. Subsequently, we performed the expression analysis and correlation analysis for potential hub genes. We determined the key genes of diabetic vasculopathy by overlapping three methods, including receiver operating characteristic (ROC) validation, least absolute shrinkage and selection operator (LASSO) model, and random forest (RF) model. We also constructed mRNA–miRNA and mRNA–TF networks, conducted immune infiltration analysis, and performed single‐cell RNA sequencing (scRNA‐seq) analysis. Finally, we performed the experimental study via construction of T2DM mouse model to further validate the accuracy of the above integrated bioinformatics analysis.

## 2. Materials and Methods

### 2.1. Acquisition of Datasets and Differential Expression Analysis

**Figure 1:**
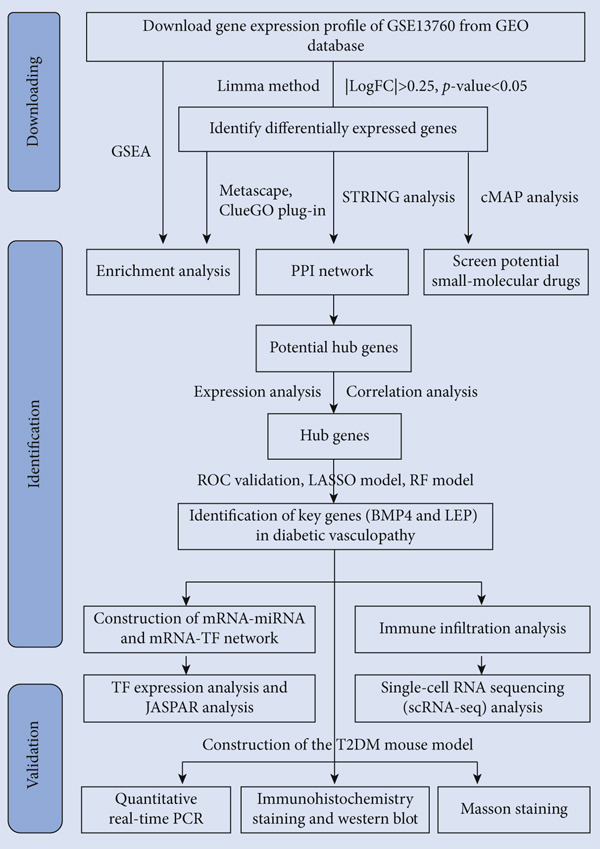
Flowchart of data analysis in this study. GEO, Gene Expression Omnibus; PPI, protein–protein interaction; GSEA, gene set enrichment analysis; ROC validation, receiver operating characteristic validation; LASSO model, least absolute shrinkage and selection operator model; RF model, random forest model; BMP4, bone morphogenetic protein 4; mRNA, message RNA; miRNA, microRNA; TF, transcription factor; T2DM, Type 2 diabetes milieus.

The GSE13760 dataset profiling diabetic arterial diseases was obtained from the Gene Expression Omnibus (GEO) database. The dataset GSE13760 was derived from arteria mammaria interna of 10 T2DM patients and 11 Con patients undergoing artery by‐pass graft surgery, which was performed by the GPL571 platform (Affymetrix Human Genome U133A 2.0 Array) (Figure 1). The baseline information between the two groups has been reported [5]. We assessed the gene expression profile and identified the DEGs between T2DM samples and Con samples based on the log_2_FC expression via limma. To avoid excessively eliminating possible DEGs, a relatively low cut‐off value of *p* value < 0.05 and |log2FC| > 0.25 was set. The heatmap of the Top 20 DEGs and the volcano plot for DEGs were displayed by the R packages of “ggplot2” (Version: 3.4.2).

### 2.2. Enrichment Analysis

In this study, multiple enrichment analyses were done with GSEA in R (Version 4.3.0), enrichment analysis in online Metascape (https://metascape.org/gp/index.html#/main/step1), and the Gene Ontology (GO) and Kyoto Encyclopedia of Genes and Genomes (KEGG) pathway enrichment analyses in ClueGo (Version 2.5.8). GSEA, a useful computational method for interpreting genome‐wide expression profiles, could determine whether a priori defined sets of genes show statistically significant, as well as concordant differences between two biological states (such as experimental and control samples), thus evaluating a significant up‐ or downregulated trend of predefined gene set [6]. In this study, GSEA was performed with the “clusterProfiler” package (Version 4.8.1), and KEGG pathways were employed to enrich gene sets. Moreover, we limited the size of gene sets ranging from 10 to 500 with *p* < 0.05 as the cutoff. We ranked the output results depending on normalized enrichment score (NES), visualizing with the “enrichplot” package (Version 1.20.0) and “ggplot2” package (Version 3.4.2).

Metascape, a biologist‐oriented online tool for analyzing system‐level datasets, illustrates the relationship between the enrichment terms. A network plot was performed with each node representing an enriched term and colored by its cluster ID. Moreover, DisGeNET and TRRUST databases in Metascape were analyzed to display the roles of DEGs in arterial pathophysiological changes between T2DM and Con samples [7, 8]. The ClueGO, a well‐known Cytoscape plug‐in, could analyze and visualize the nonredundant biological terms, including GO and KEGG enrichment, with large clusters of genes among a functionally grouped network [9].

### 2.3. cMAP Analysis

The cMAP, a gene expression profile database based on the intervention of gene expression signatures, could illustrate the relationships between genes, diseases, and small‐molecule compounds (https://clue.io) [10, 11]. In our study, upregulated DEGs were analyzed in the cMAP online database to identify the potential small‐molecular drugs for diabetic vasculopathy treatment. Finally, the Top 10 small‐molecular compounds with the highest negative enrichment scores were screened.

### 2.4. Generation of a PPI Network and Screening for Hub Genes

The PPI network was constructed utilizing the STRING database, a comprehensive repository of known and predicted protein interactions, encompassing both direct (physical) and indirect (functional) associations. This approach facilitates the elucidation of disease‐related mechanisms. Based on the STRING online analysis results, the CytoHubba in Cytoscape (Version 3.7.1) was used to screen the potential hub genes. For the plug‐in CytoHubba, four algorithms, such as gene connection degree, density of maximum neighborhood component (DMNC), maximum neighborhood component (MNC), and maximal clique centrality (MCC), were applied, and the Top 20 genes were screened, respectively. The potential hub genes were screened by overlapping the Top 20 genes of four CytoHubba algorithms, which were displayed using the Venn tool.

### 2.5. The Expression Analysis and Correlation Analysis of Potential Hub Genes

The expression analysis of the potential hub genes between T2DM and Con samples was performed using Student′s *t*‐test from GSE13760, which was visualized with GraphPad Prism software (Version 7.00). Moreover, the correlation analysis of potential hub genes was conducted using the “corrplot” package (Version 0.92) and visualized with the “circlize” package (Version 0.4.15) and the “GGally” package (Version 2.1.2) in R (Version 4.3.0). Meanwhile, the Top 3 positive and negative correlations between hub genes were visualized using the “ggstatsplot” package (Version 0.11.1).

### 2.6. Identification of the Key Genes in Diabetic Vasculopathy

In this study, a total of three methods were applied to identify the key genes of diabetic vasculopathy, including ROC validation, LASSO model, and RF model [12, 13]. The ROC validation could evaluate the diagnostic value of the potential hub genes for DCM. The area under the ROC curve (AUC) > 0.7 with *p* < 0.05 indicated good discrimination. The LASSO and RF were two classical algorithms of machine learning. The LASSO algorithm could filter variables to enhance the predictive performance to identify the candidate genes and establish a diagnostic model for diabetic vasculopathy with the “glmnet” package (Version 4.1‐7). The RF algorithm could integrate multiple trees through the idea of ensemble learning to gain better accuracy, subsequently narrowing down the candidate genes with the “randomForest” package (4.7‐1.1). Finally, the overlapping genes of the ROC validation, LASSO model, and RF model were considered as the key genes of diabetic vasculopathy.

### 2.7. Construction of mRNA–miRNA, mRNA–TF Network, and JASPAR Analysis

To comprehensively identify the potential regulatory relationships between mRNAs and miRNAs, as well as mRNAs and TFs in diabetic vasculopathy, the targeted miRNAs of key genes were based on the miRWalk and miRDB databases [14, 15]. The target TFs of key genes in diabetic vasculopathy were predicted according to the hTFtarget and knockTF databases [16, 17]. miRNAs identified in both databases were defined as targeted miRNAs of key genes. The targeted miRNAs and TFs of key genes in diabetic vasculopathy were determined by overlapping both databases, respectively. The mRNA–miRNA, as well as mRNA–TF interaction networks were constructed using Cytoscape (Version 3.7.1). Moreover, we performed expression analysis for target TFs using GSE13760 and visualized them using the “ggplot2” package (Version: 3.4.2).

In addition, the JASPAR database is an open‐access database that provides comprehensive information on transcription factor binding sites (http://jaspar.genereg.net/). The base sequence of the potential promoter region of the target gene was obtained from the NCBI database (https://www.ncbi.nlm.nih.gov/). In general, for each target gene, 2000 nucleotides have been analyzed: from 2000 upstream to the transcription starting site. Predicting the sequences of binding sites of candidate transcription factors in the promoter region of the target gene was screened in the JASPAR database with the relative profile score threshold of 80%.

### 2.8. Immune Infiltration Analysis

To better identify the immune cell characteristics between T2DM samples and Con samples, we compared the differences in immune cell subsets in the samples, which were presented with the heatmap using the “ComplexHeatmap” package (Version 2.16.0). Meanwhile, we compared the differential composition of 28 immune cells between the two groups using the single sample gene set enrichment analysis (ssGSEA) with the “ggplot” package (Version 3.4.2). Spearman′s correlation analysis was performed with the 28 types of infiltrating immune cells using the “Corrplot” package (Version 0.92). The Pearson correlation analysis was used to evaluate the correlation between the two key genes in diabetic vasculopathy and the distribution of immune cells with “pheatmap” package (Version 1.0.12).

### 2.9. scRNA‐Seq Analysis

scRNA‐seq data of aortic tissue specimens from control and diabetic mice were obtained from the NCBI GEO database (http://www.ncbi.nlm.nih.gov/geo/, Accession Number: GSE211216) and the Single Cell Portal (https://singlecell.broadinstitute.org/single_cell, Study ID: SCP1361). The scRNA‐seq data were processed using the R package “Seurat” (Version 4.4.1) following established protocols. Briefly, low‐quality cells were excluded based on predefined quality control criteria as described in the original studies. The data were then normalized and scaled, followed by principal component analysis (PCA) to reduce dimensionality. Highly variable genes were identified, and clustering analysis was performed using the Seurat pipeline. Uniform Manifold Approximation and Projection (UMAP) was applied for further dimensionality reduction and visualization of cell populations. DEGs between experimental groups were identified using the “FindMarkers” function in Seurat, with a significance threshold of *p* < 0.05. Genes exhibiting an average log2FC > 0 and an adjusted *p* value < 0.05 were selected for functional enrichment analysis. GO and KEGG pathway analyses were conducted using the R package “clusterProfiler” (Version 4.8.1) to elucidate the biological processes (BPs) and pathways associated with the identified DEGs.

### 2.10. Construction of the T2DM Mouse Model

C57BL/6N male mice (6–8 weeks) were acquired from Changzhou Cavins Laboratory Animal Co. Ltd. We randomized the mice into two groups after 1 week of acclimatization, including the Con group and T2DM group (*n* = 10 per group). The Con group was fed with a normal diet, and the T2DM group received a high‐fat diet (45% fat and 1% cholesterol). After 4 weeks of feeding, the T2DM group mice were treated intraperitoneally with 40 mg/kg of streptozotocin (STZ) dispersed in trisodium citrate solution (pH 4.5). The control mice were treated with citrate buffer. After 12 weeks, T2DM mice were determined when the blood glucose ranged from 11.1 to 28.0 mM. Moreover, an intraperitoneal glucose tolerance test (IPGTT) was performed to assess glucose tolerance at 6 weeks. In this study, the thoracic aorta was collected for tissue preparation. Animal experiments were approved by the Institutional Animal Care and Use Committee of Soochow University (SYXK(SU)2021‐0065).

### 2.11. Quantitative Real‐Time PCR (qPCR)

Total RNA was isolated from the murine thoracic aorta. Complementary DNA (cDNA) was then synthesized from the extracted RNA using a commercial reverse transcription kit (Takara, DRR036A). Subsequent qPCR was performed on an ABI Prism 7500HT instrument (Applied Biosystems) with SYBR Green master mix (Roche, 4913914001). Gene expression levels were normalized to the endogenous control *β*‐actin to account for variations in initial RNA input. The sequences of the primer pairs employed are listed as follows: CCATACCTTGACCCGCAGAA (forward) and AATGGCGACGGCAGTTCTTA (reverse) for BMP4, ATTTCACACACGCAGTCGGT (forward) and ACTCAGAATGGGGTGAAGCC (reverse) for LEP, and AGATTACTGCCCTGGCTCCTA (forward) and CCTGCTTGCTGATCCACATCT (reverse) for *β*‐actin.

### 2.12. Immunohistochemistry Staining

For detection of BMP4 using the immunohistochemical method, fixed mouse thoracic aorta with 4% paraformaldehyde was dehydrated in a decreasing series of alcohol concentrations. After embedding in paraffin, mouse aortas were cut into 5 *μ*m sections. The paraffin section was treated with endogenous peroxidase and blocked using 5% goat serum at room temperature. The section was incubated with primary antibody against BMP4 (Abcam, ab155033; 1/400) at 4°C overnight. The section was then incubated using a biotinylated secondary antibody (Zsbio Store, SP‐900; ZLI‐9018) for 60 min at room temperature. Finally, all sections were counterstained with hematoxylin staining solution.

### 2.13. Western Blot

Arterial tissues from T2DM and Con mouse thoracic aorta were taken from −80°C storage and homogenized and soaked in RIPA buffer (Thermo Fisher Scientific Co., #89901) containing phosphatase inhibitor and protease inhibitor (Thermo Fisher Scientific Co., #78442). BCA Protein Assay Kit (Thermo Fisher Scientific Co., #23227) was used to verify the protein concentration, as well as to adjust it to a uniform level. The PVDF membranes were incubated with primary antibodies against BMP4 (Abcam, ab155033; 1/1000) and *β*‐tubulin (Abcam, ab21058; 1/3000) at 4°C overnight, followed by corresponding secondary antibody incubation. Immunoblot bands were quantified with ImageJ software (Scion Corp., United States).

### 2.14. Masson Staining

A 5‐*μ*m section of arterial tissue was stained with Masson trichrome staining solution (#D026, Nanjing Jiancheng Bioengineering Institute). The sections were taken under a 10x and 40x objective lens. We used Image‐Pro Plus for the quantification of fibrosis areas (Media Cybernetics Inc.).

### 2.15. Statistical Analysis

Statistical analysis was conducted via R (Version 4.3.0), GraphPad Prism 7, and SPSS 25.0. Data are displayed as mean ± SEM. Moreover, Student′s *t*‐test was used to compare data between the T2DM and Con group. A threshold of *p* < 0.05 was used to define statistical significance.

## 3. Results

### 3.1. Identification of the DEGs Between T2DM and Con Samples

From the GSE13760 dataset, 139 genes were identified as differentially expressed (29 upregulated and 110 downregulated). To visualize the results, Figure 2a depicts a heatmap of the Top 20 DEGs in each direction. The volcano plot in Figure 2b graphically summarizes the fold‐change and statistical significance for all analyzed genes.

Figure 2Identification of DEGs in T2DM samples. (a) The expression heatmap of the Top 20 upregulated and downregulated DEGs between T2DM and Con samples. (b) The expression volcanic plots of the differential gene expression between T2DM and Con samples. DEGs, differentially expressed genes; T2DM, Type 2 diabetes milieus; Con, control.

**Figure:**
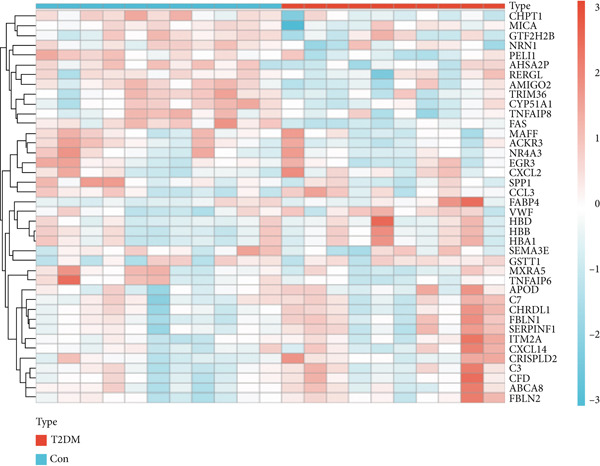
(a)

**Figure:**
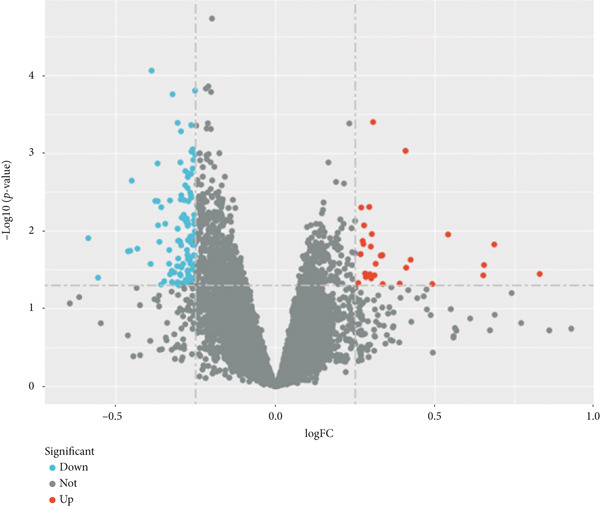
(b)

### 3.2. Functional Analysis

GSEA based on all DEGs without limitation of *p* value and log_2_FC was performed, and the results showed that cytokine–cytokine receptor interaction, ECM‐receptor interaction, nuclear factor‐kappa B (NF‐*κ*B) signaling pathway, TNF signaling pathway, and T2DM were positively correlated with the T2DM sample (Figure 3a), while the citrate cycle (TCA cycle), nucleotide excision repair, pyruvate metabolism, ubiquitin‐mediated proteolysis, valine, leucine, and isoleucine degradation were positively correlated with the Con sample (Figure 3b).

Figure 3GSEA for all DEGs in T2DM samples. (a) Five enrichment items were positively correlated with T2DM samples. (b) Five enrichment items were positively correlated with Con samples. GSEA, gene set enrichment analysis; DEGs, differentially expressed genes; T2DM, Type 2 diabetes milieus; Con, control.

**Figure:**
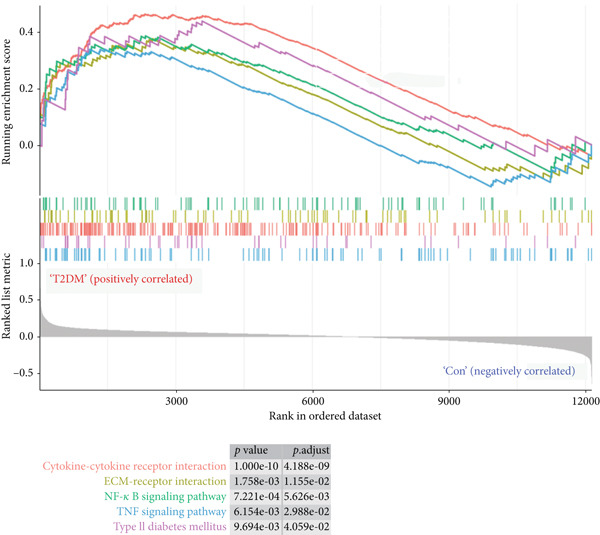
(a)

**Figure:**
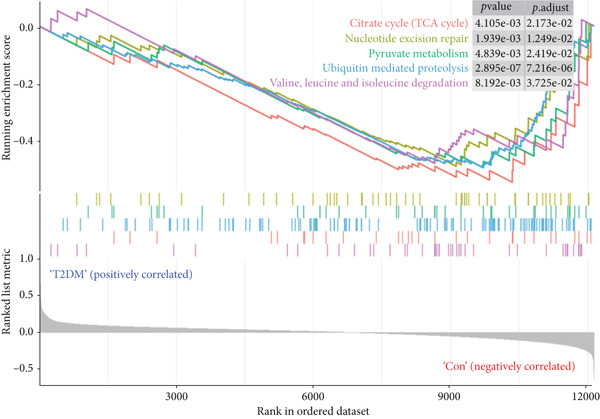
(b)

The functional analysis for the 139 DEGs from the Metascape showed that the Top 20 clusters of the signal pathway and BP were enriched, including small molecule biosynthetic process, aortic valve morphogenesis, NABA ECM glycoproteins, regulation of MAPK cascade, and programmed cell death (Figure 4a). Moreover, DisGeNET analysis suggested that multiple cardiovascular‐related diseases, such as ascending aorta dilatation, prehypertension, postmyocardial infarction, idiopathic pulmonary arterial hypertension, and hyperlipidemia, were significantly associated with the 139 DEGs between T2DM and Con samples (Figure 4b). TRRUST analysis also revealed that the Top 8 transcription factors, including EGR, NFIC, YBX1, SP1, HIF1A, TFAP2A, STAT3, and STAT1, were associated with the 139 DEGs (Figure 4c).

Figure 4Functional analysis for 139 DEGs in T2DM samples. (a) Metascape analysis showed the Top 20 clusters of the signal pathway and biological process. (b) DisGeNET analysis for the 139 DEGs between T2DM and Con samples. (c) TRRUST analysis for the 139 DEGs between T2DM and Con samples. (d) GO analysis with biological processes for the 139 DEGs based on the ClueGO plug‐in. (e) GO analysis with cellular components for the 139 DEGs based on the ClueGO plug‐in. (f) GO analysis with molecular functions for the 139 DEGs based on the ClueGO plug‐in. (g) KEGG analysis for the 139 DEGs based on the ClueGO plug‐in. (h) The expression heatmap of TGF‐beta signaling pathway in KEGG analysis. (i) The expression heatmap of leukocyte transendothelial migration pathway in KEGG analysis. DEGs, differentially expressed genes; T2DM, Type 2 diabetes milieus; Con, control; GO, Gene Ontology; KEGG, Kyoto Encyclopedia of Genes and Genomes.

**Figure:**
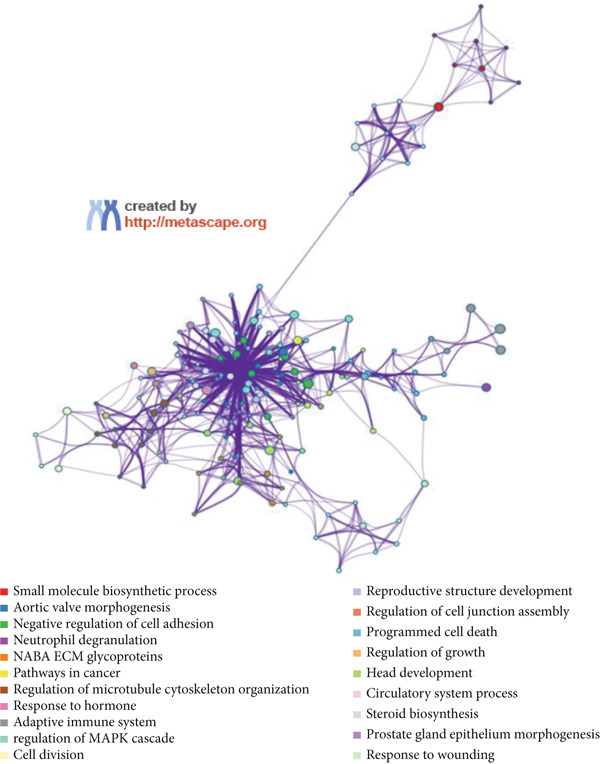
(a)

**Figure:**
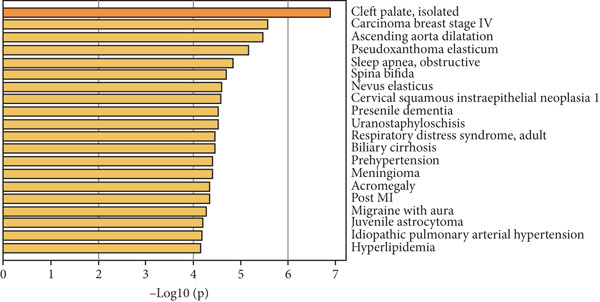
(b)

**Figure:**
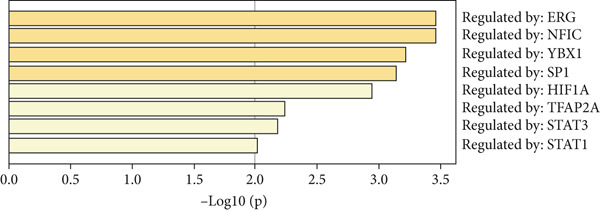
(c)

**Figure:**
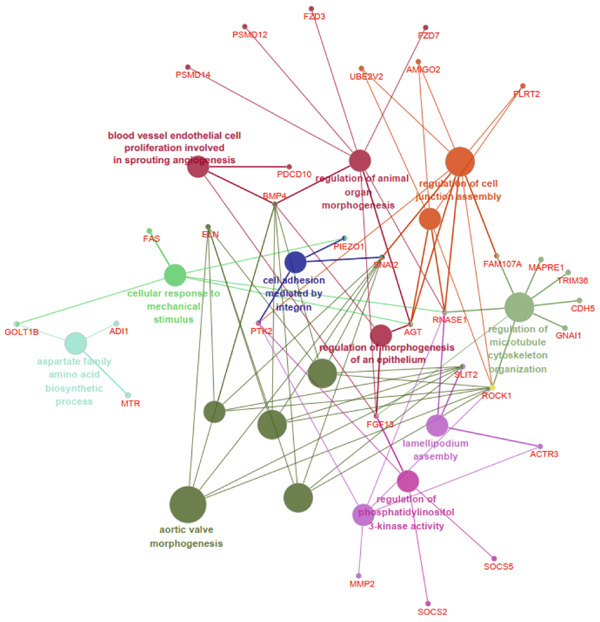
(d)

**Figure:**
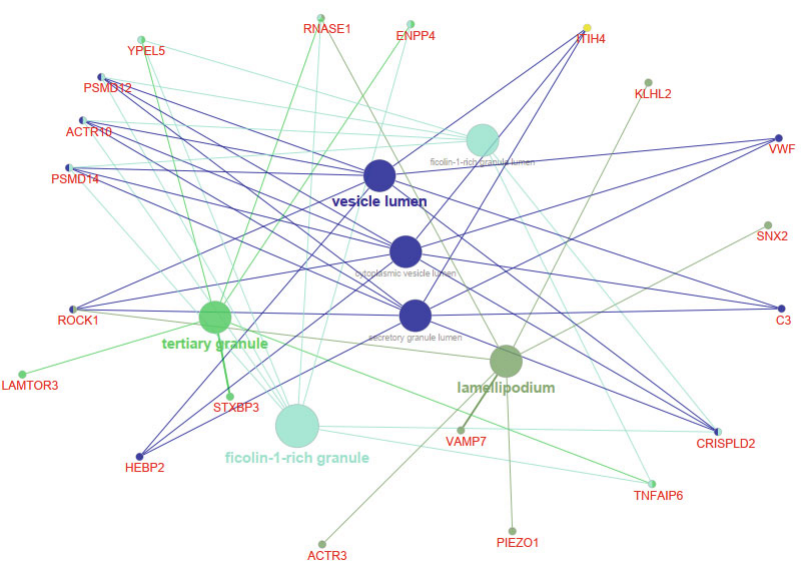
(e)

**Figure:**
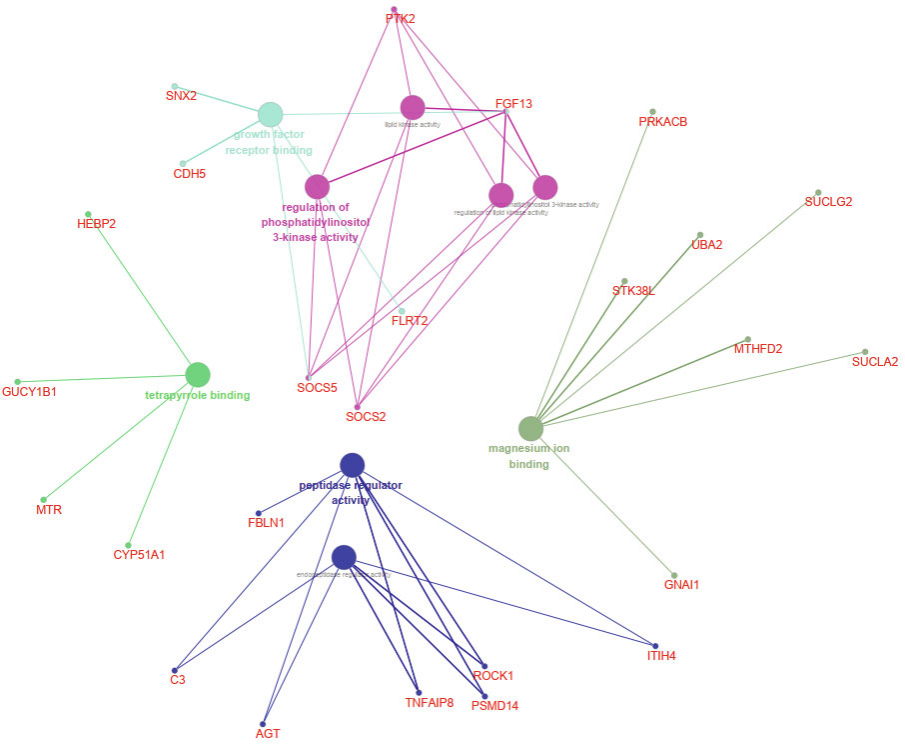
(f)

**Figure:**
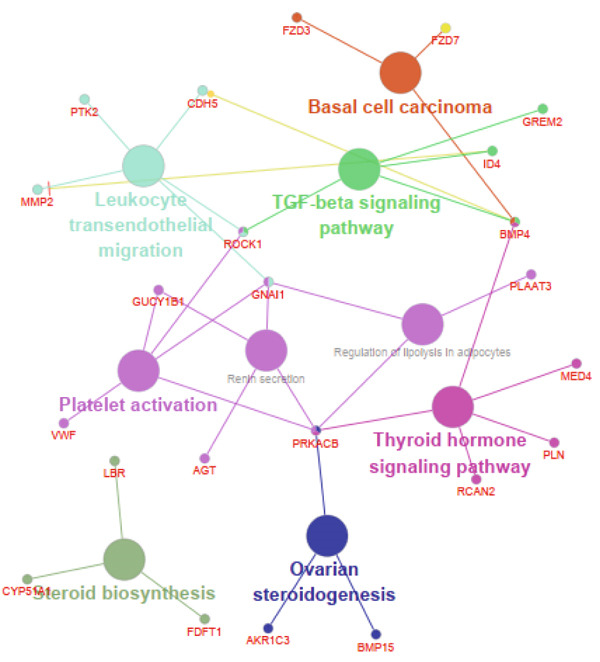
(g)

**Figure:**
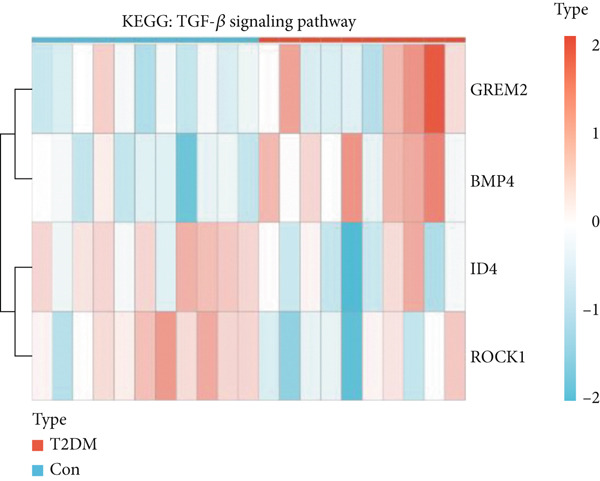
(h)

**Figure:**
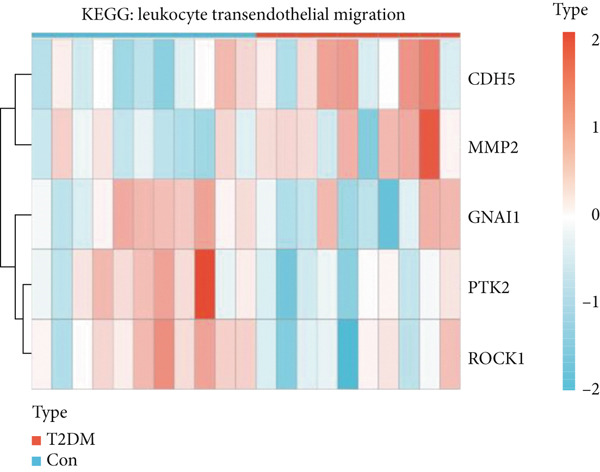
(i)

In addition, the GO analysis for the 139 DEGs based on the ClueGO plug‐in was performed. The enriched BPs were mainly associated with regulation of phosphatidylinositol 3‐kinase activity (GO: 0043551), blood vessel endothelial cell proliferation involved in sprouting angiogenesis (GO:0002043), regulation of cell junction assembly (GO:1901888), aortic valve morphogenesis (GO:0003180), and cellular response to mechanical stimulus (GO:0071260) (Figure 4d and Supporting Information 2: Table S1 and Supporting Information 1: Figure S1). The cellular components (CCs) were mainly related to lamellipodium (GO:0030027), ficolin‐a‐rich granule (GO:0101002), tertiary granule (GO:0070820), and vesicle lumen (GO:0031983) (Figure 4e and Supporting Information 3: Table S2 and Supporting Information 1: Figure S2). Enriched molecular functions (MFs) mainly involved in phosphatidylinositol 3‐kinase activity (GO:0035004), growth factor receptor binding (GO:0070851), tetrapyrrole binding (GO:0046906), magnesium ion binding (GO:0000287), and peptidase regulator activity (GO:0061134) (Figure 4f and Supporting Information 4: Table S3 and Supporting Information 1: Figure S3).

Meanwhile, KEGG pathway enrichment analysis mainly included transforming growth factor‐beta (TGF‐*β*) signaling pathway (KEGG:04350), leukocyte transendothelial migration (KEGG:04670), platelet activation (KEGG:04670), thyroid hormone signaling pathway (KEGG:04919), and steroid biosynthesis (KEGG:00100) (Figure 4g and Supporting Information 5: Table S4 and Supporting Information 1: Figure S4). Importantly, TGF‐*β* signaling pathway included four DEGs (*GREM2*, *BMP4*, *ID4*, and *ROCK1*), and leukocyte transendothelial migration pathway included five DEGs (*CHD5*, *MMP2*, *GNAI1*, *PTK2*, and *ROCK1*), which were illustrated by heatmap (Figure 4h,i), respectively.

### 3.3. Screening the Potential Small‐Molecular Compounds for Diabetic Vasculopathy Therapy

In this study, the cMAP database was used to screen the candidate small‐molecular drugs, which could reverse the altered expression of diabetic vasculopathy genes and exert a therapeutic effect in diabetic vasculopathy patients. A total of 29 upregulated DEGs were imported into the cMAP database to identify the small‐molecule compounds. Following the significantly detailed inquiry, the Top 10 small‐molecule compounds, including diazepam, HEAT, methantheline, PT‐630, deferiprone, doxercalciferol, androstenedione, flupirtine, VX‐222, and NSC‐94258, with the highest negative scores were determined to be potential pharmacological therapeutic agents for diabetic vasculopathy treatment (Figure 5a).

Figure 5Screening the potential small‐molecular compounds for diabetic vasculopathy therapy based on cMAP database. (a) The Top 10 small‐molecule compounds were identified based on 29 upregulated DEGs. (b) The Sankey plot showed the descriptions of the targeted pathways for the 10 candidate small‐molecule compounds. (c) The chemical structures of the 10 candidate small‐molecule compounds. DEGs, differentially expressed genes.

**Figure:**
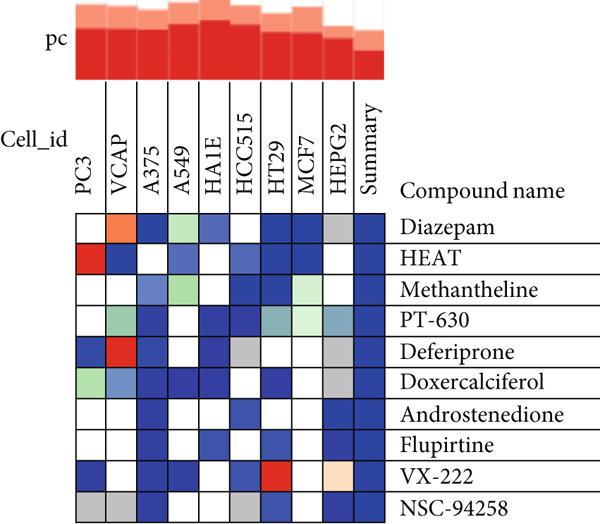
(a)

**Figure:**
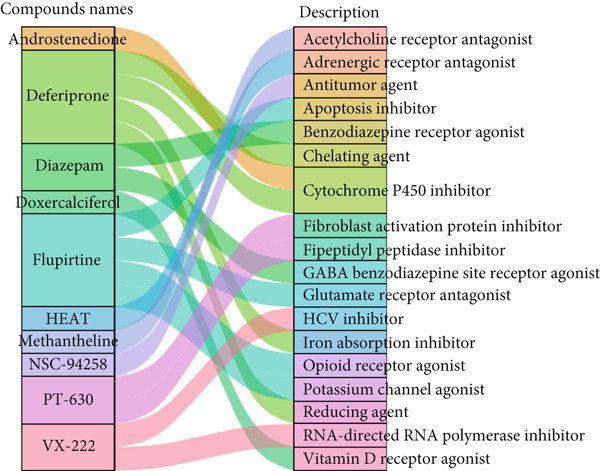
(b)

**Figure:**
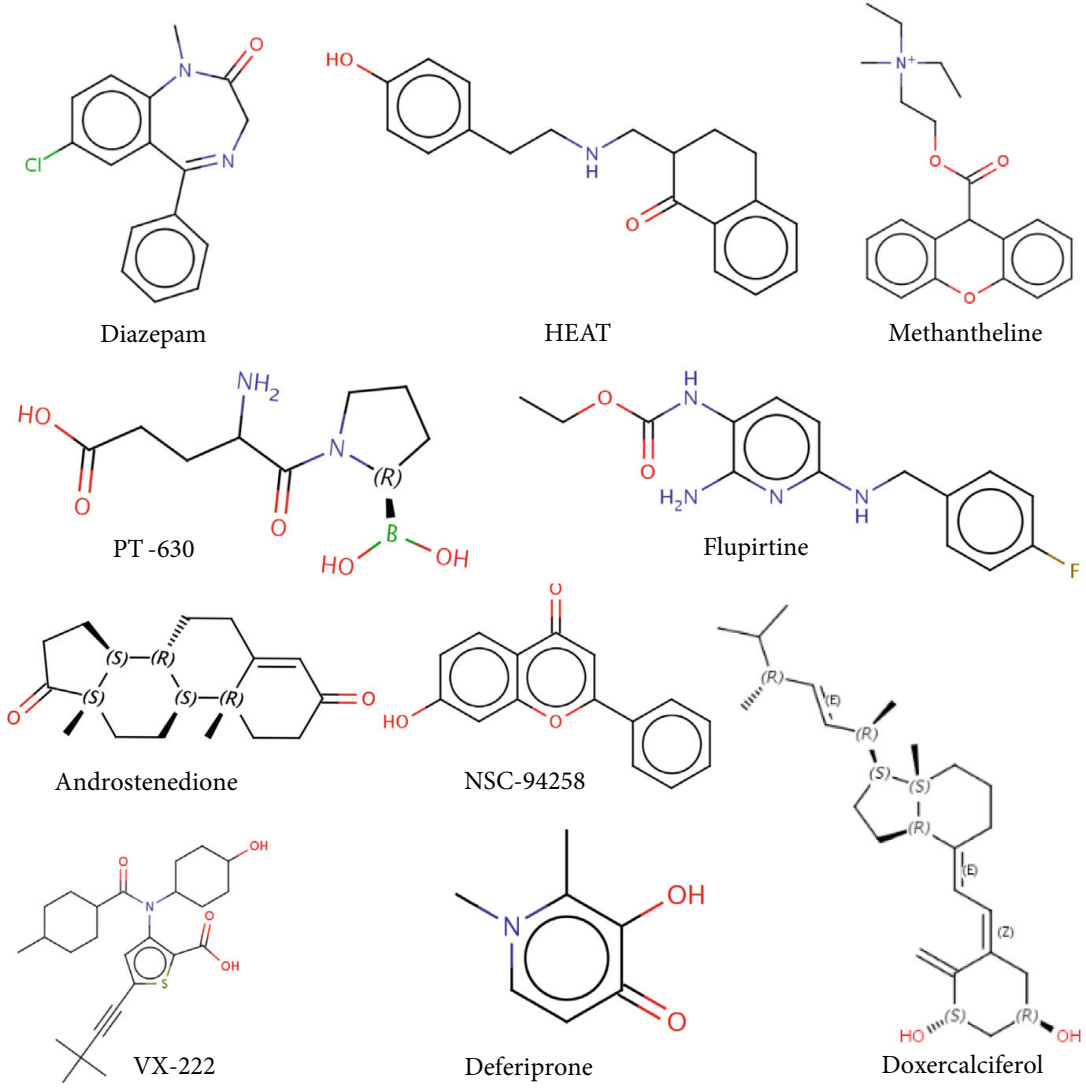
(c)

The descriptions of the targeted pathways for the 10 candidate small‐molecule compounds included benzodiazepine receptor agonist, GABA benzodiazepine site receptor agonist, adrenergic receptor antagonist, acetylcholine receptor antagonist, dipeptidyl peptidase inhibitor, fibroblast activation protein inhibitor, iron absorption inhibitor, chelating agent, reducing agent, cytochrome P450 inhibitor, vitamin D receptor agonist, glutamate receptor antagonist, potassium channel agonist, apoptosis inhibitor, opioid receptor agonist, HCV inhibitor, RNA‐directed RNA polymerase inhibitor, and antitumor agent (Figure 5b). Importantly, the chemical structures of these 10 compounds were illustrated in Figure 5c.

### 3.4. Identification of Potential Hub Genes

PPI network of 139 DEGs was constructed using STRING, including a total of 92 nodes and 128 edges (Figure 6a). Subsequently, the Top 20 genes, respectively, from four algorithms of CytoHubba, including degree, DMNC, MCC, and MNC, were screened, which are visualized in Figures 6b, 6c, 6d, and 6e. Finally, a total of 14 potential hub genes were identified via overlapping the Top 20 genes of four CytoHubba algorithms (Figure 6f).

Figure 6Identification of potential hub genes. (a) PPI network for 139 DEGs. The Top 20 genes, respectively, were screened from four CytoHubba algorithms, including (b) degree, (c) DMNC, (d) MCC, and (e) MNC. (f) Potential hub genes were identified via overlapping the Top 20 genes of four CytoHubba algorithms.

**Figure:**
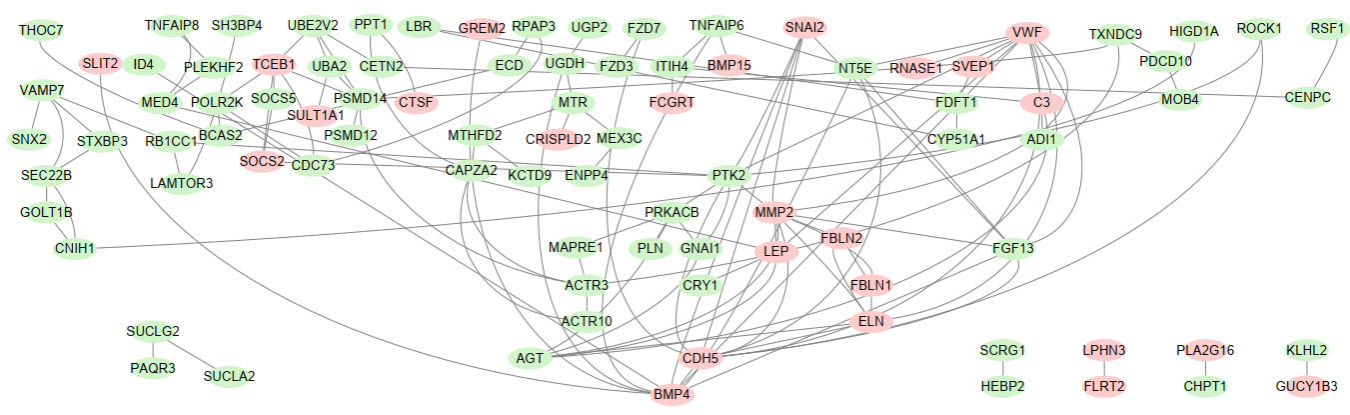
(a)

**Figure:**
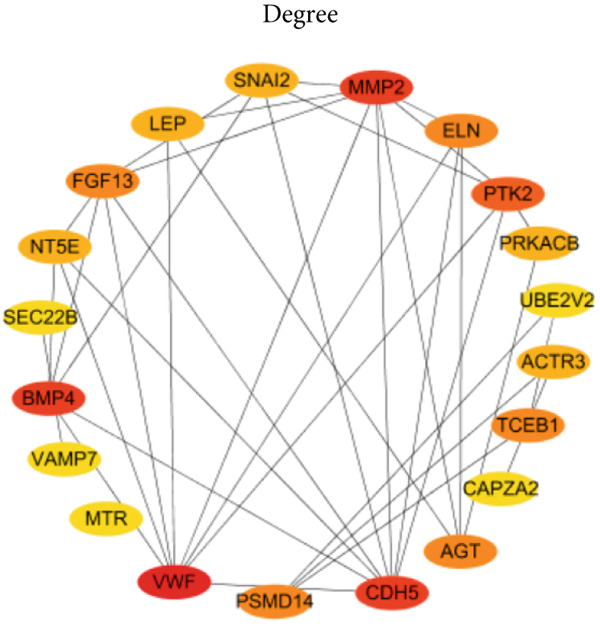
(b)

**Figure:**
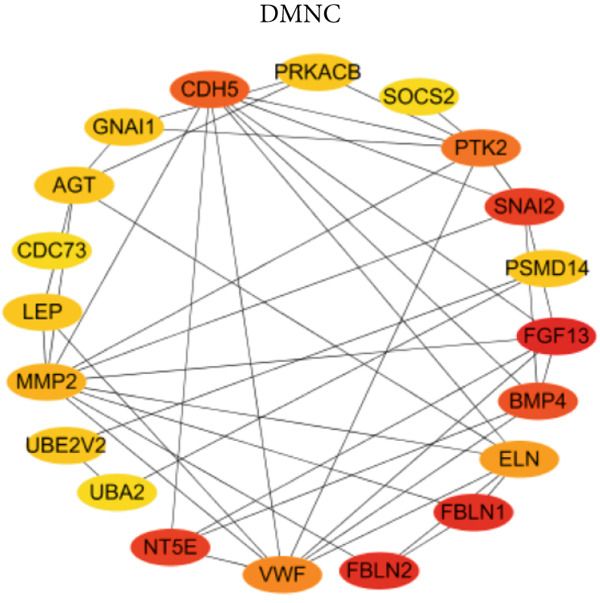
(c)

**Figure:**
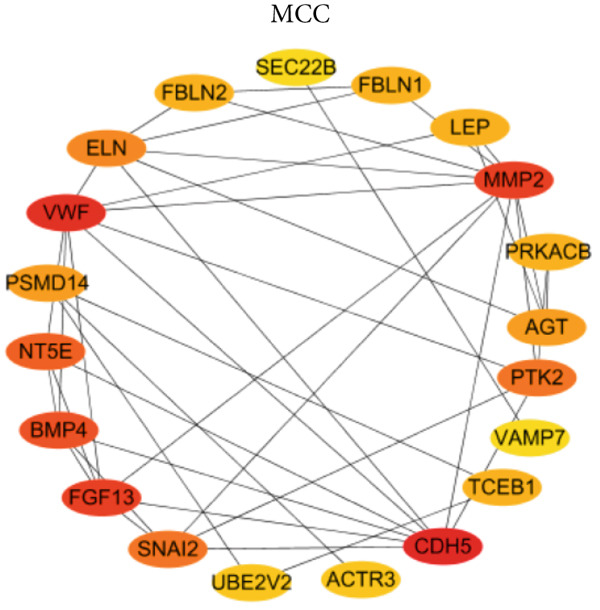
(d)

**Figure:**
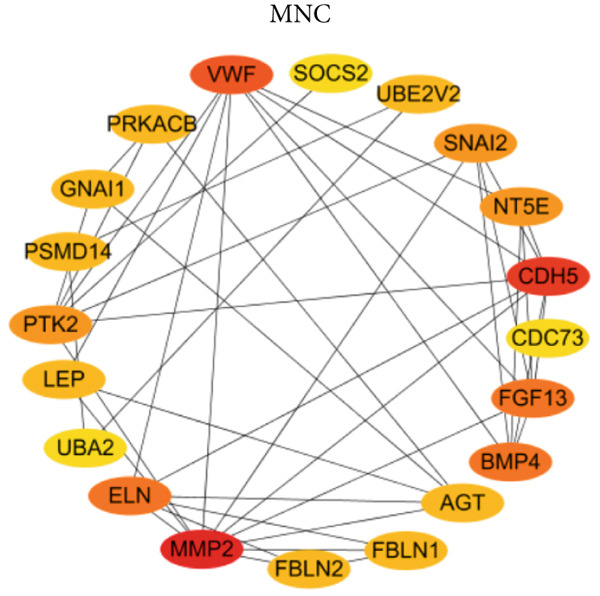
(e)

**Figure:**
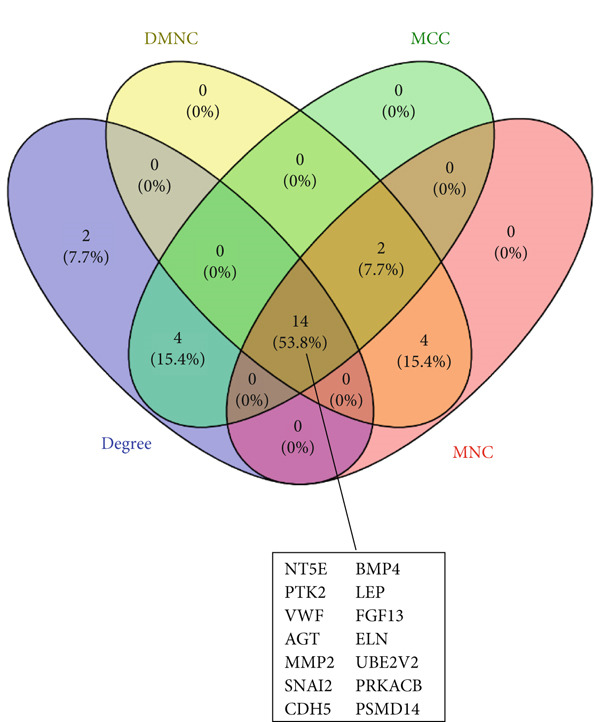
(f)

### 3.5. The Expression Analysis and Correlation Analysis of Potential Hub Genes

The expression analysis for the 14 potential hub genes was performed, and only 10 hub genes between T2DM samples and Con samples were statistically different (Figure 7a), in which four hub genes (BMP4, MMP2, SNAI2, and LEP) were significantly upregulated and six hub genes (PTK2, FGF13, NT5E, AGT, UBE2V2, and PSMD14) were significantly downregulated. Meanwhile, correlation analysis for 10 hub genes is displayed in Figure 7b,c with the correlation coefficient ranging from −0.663 to 0.843. In addition, the Top 3 positive correlation hub genes were BMP4 and MMP2 (*r* = 0.78; 95*%* *C* *I* = 0.53, 0.91; *p* = 3.04e − 05), PTK2 and FGF13 (*r* = 0.76; 95*%* *C* *I* = 0.49, 0.90; *p* = 6.01e − 05), and PSMD and UBE2V2 (*r* = 0.84; 95*%* *C* *I* = 0.65, 0.93; *p* = 1.62e − 06), respectively (Figures 7d, 7e, and 7f). The Top 3 negative correlation hub genes were BMP4 and PSMD14 (*r* = −0.66; 95*%* *C* *I* = −0.85, ‐0.32; *p* = 1.05e − 03), BMP4 and UBE2V2 (*r* = −0.63; 95*%* *C* *I* = −0.84, ‐0.28; *p* = 2.02e − 03), and MMP2 and UBE2V2 (*r* = −0.65; 95*%* *C* *I* = −0.85, ‐0.31; *p* = 1.37e − 03), respectively (Figures 7g, 7h, and 7i).

Figure 7The expression analysis and correlation analysis of potential hub genes. (a) Ten hub genes between T2DM samples and Con samples were statistically different. (b) Chord diagram and diagonal matrix diagram showed the correlation analysis for 10 hub genes. (d–f) The Top 3 positive correlation hub genes. (g–i) The Top 3 negative correlation hub genes. T2DM, Type 2 diabetes milieus; Con, control.

**Figure:**
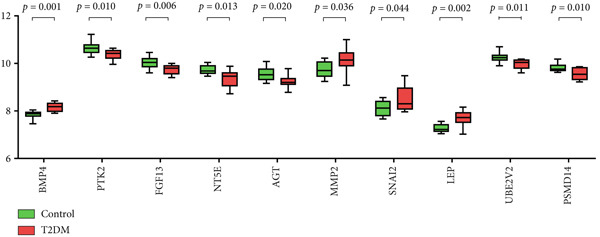
(a)

**Figure:**
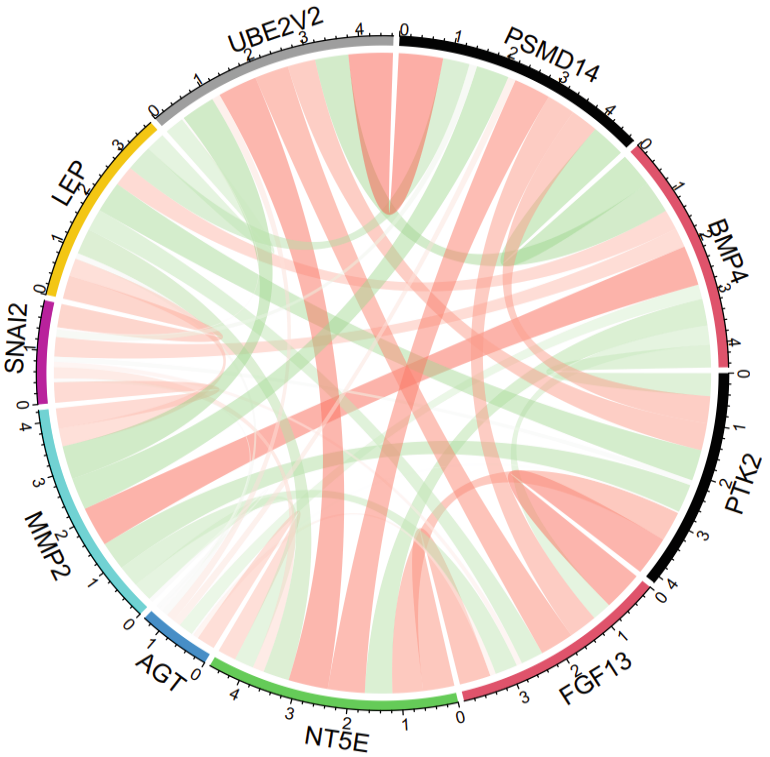
(b)

**Figure:**
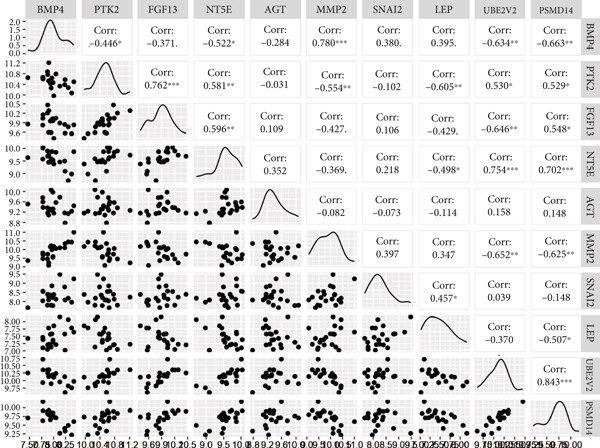
(c)

**Figure:**
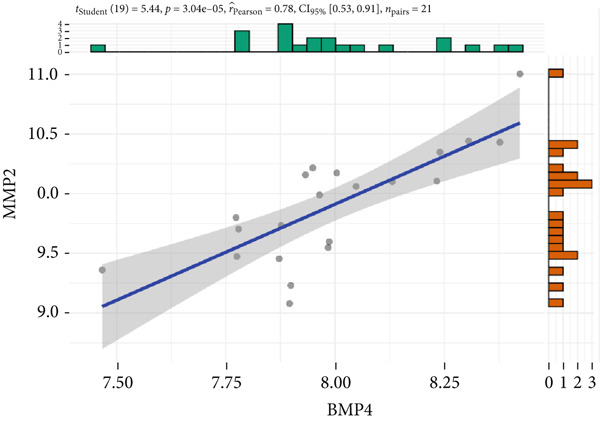
(d)

**Figure:**
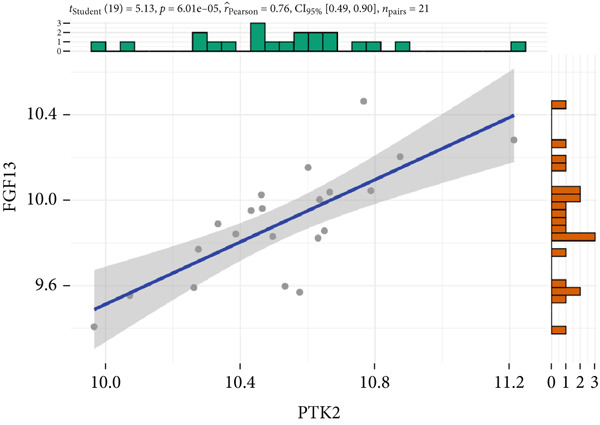
(e)

**Figure:**
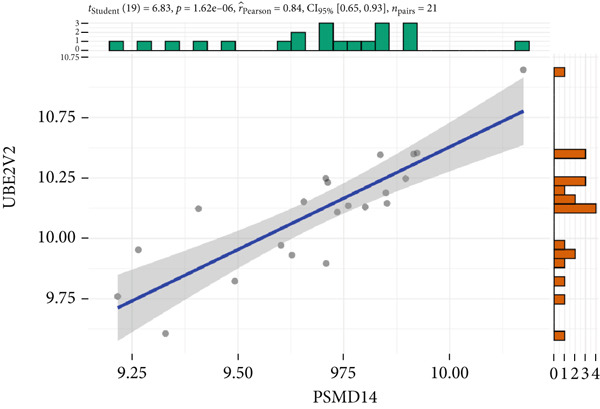
(f)

**Figure:**
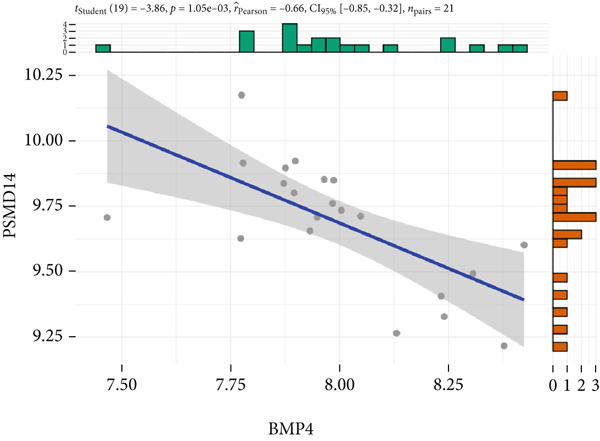
(g)

**Figure:**
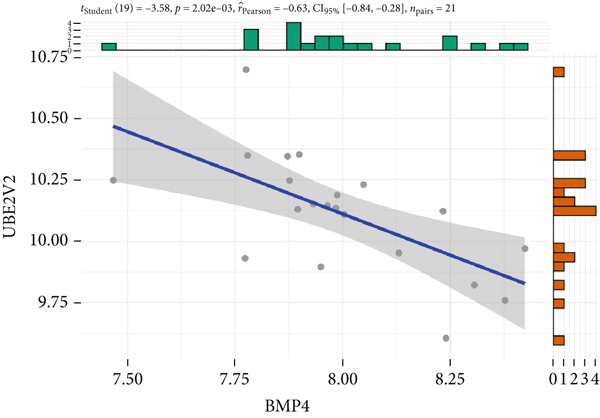
(h)

**Figure:**
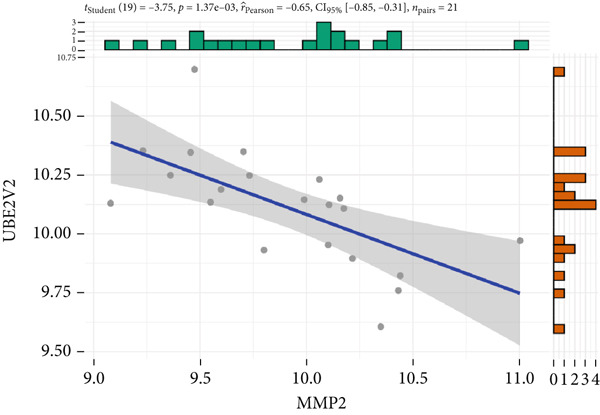
(i)

### 3.6. Identification of the Key Genes in Diabetic Vasculopathy

**Figure 8:**
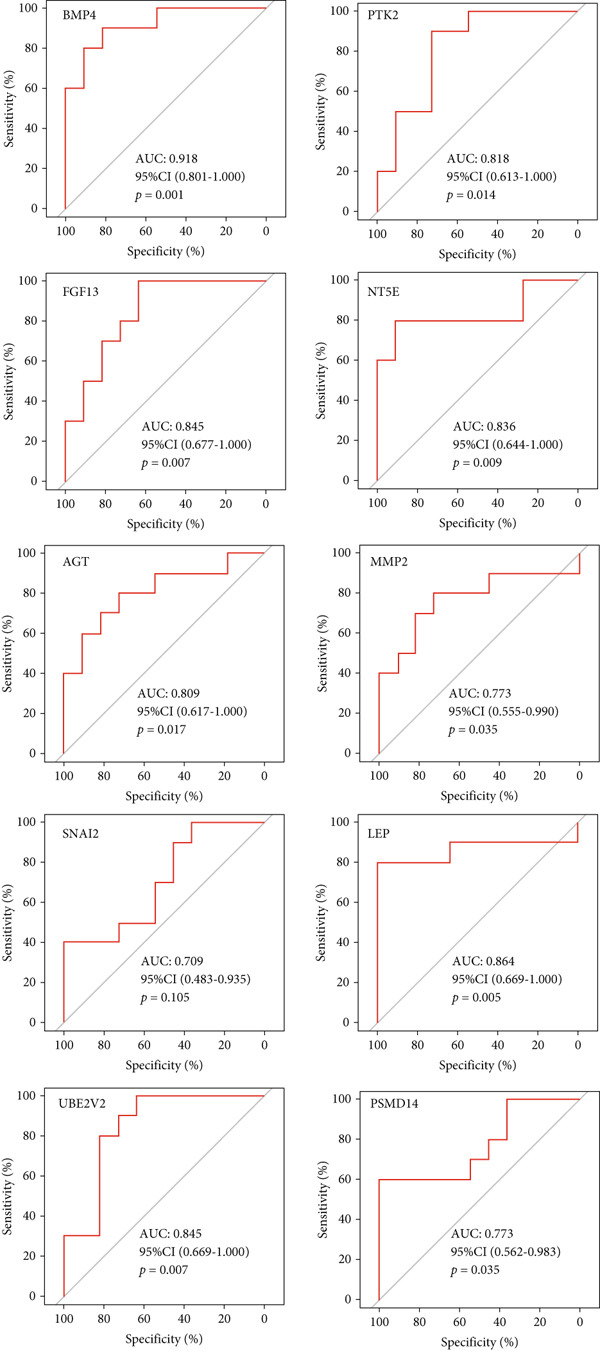
The ROC validation for the 10 hub genes. The ROC validation for the 10 hub genes was performed and nine hub genes showed AUCs > 0.7 with *p* < 0.05 (including 0.918 for BMP4, 0.818 for PTK2, 0.845 for FGF13, 0.836 for NT5E, 0.809 for AGT, 0.773 for MMP2, 0.864 for LEP, 0.845 for UBE2V2, and 0.773 for PSMD14, respectively). ROC validation, receiver operating characteristic validation.

A total of three methods were applied to identify the key genes of diabetic vasculopathy. The ROC validation for the 10 hub genes was performed, and nine hub genes showed AUCs > 0.7 with *p* < 0.05 (including 0.918 for BMP4, 0.818 for PTK2, 0.845 for FGF13, 0.836 for NT5E, 0.809 for AGT, 0.773 for MMP2, 0.864 for LEP, 0.845 for UBE2V2, and 0.773 for PSMD14, respectively) (Figure 8), indicating that the hub genes had a good discrimination for T2DM samples from Con samples. LASSO regression algorithm was applied to identify five potential candidate genes (BMP4, FGF13, AGT, SNAI2, and LEP) from 10 hub genes, which have a significant impact on the diagnosis of diabetic vasculopathy patients (Figures 9a, 9b, and 9c). Meanwhile, a personalized nomogram prediction model was constructed based on the five potential candidate genes (Figure 9d), and the nomogram showed good discrimination with an AUC of 1.000 (Figure 9e). The Hosmer–Lemeshow goodness‐of‐fit test with a mean absolute error of 0.068 indicated that the nomogram has a good calibration (Figure 9f), and the decision curve analysis (DCA) suggested that the nomogram has good clinical applicability (Figure 9g). Additionally, the RF machine learning algorithm was also used to sequence 10 hub genes according to the variable importance of each gene, and the Top 2 genes with the MeanDecreaseGini > 1 were screened (Figure 9h,i). Finally, two genes (BMP4 and LEP) were considered as the key genes of diabetic vasculopathy via overlapping genes of ROC validation, LASSO model, and RF model (Figure 9j).

Figure 9Identification of the key genes in diabetic vasculopathy. (a–c) LASSO regression algorithm was applied to identify five potential candidate genes. (d) A personalized nomogram prediction model was constructed based on the five potential candidate genes. (e) The nomogram showed good discrimination with AUC of 1.000. (f) The Hosmer–Lemeshow goodness‐of‐fit test. (g) DCA suggested that the nomogram has good clinical applicability. (h, i) RF algorithm was used to sequence 10 hub genes. (j) Identification of key genes of diabetic vasculopathy via overlapping genes of ROC validation, LASSO model, and RF model. DCA, decision curve analysis; ROC validation, receiver operating characteristic validation; LASSO model, least absolute shrinkage and selection operator model; RF model, random forest model.

**Figure:**
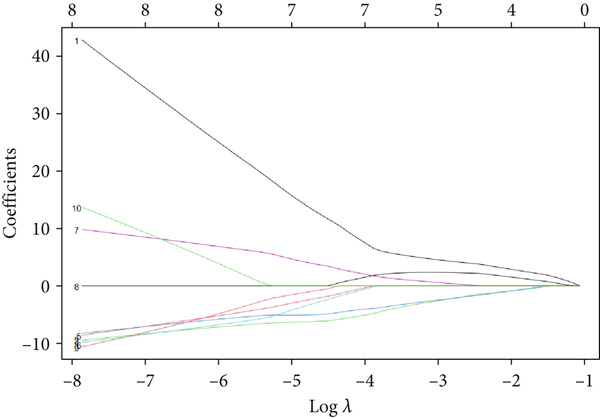
(a)

**Figure:**
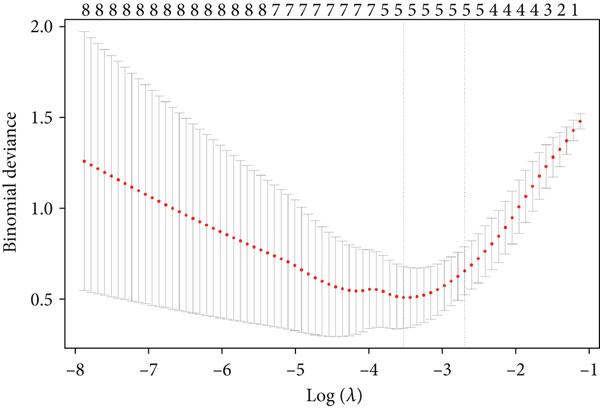
(b)

**Figure:**
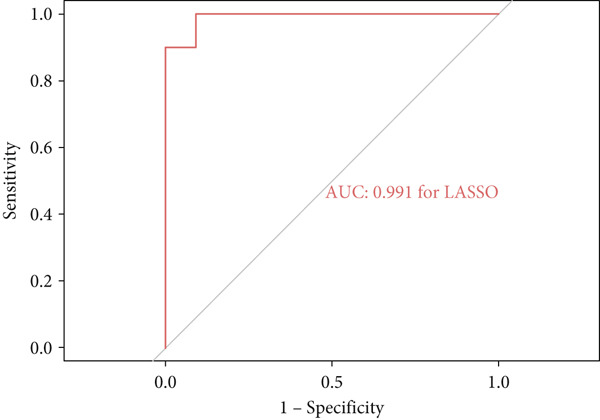
(c)

**Figure:**
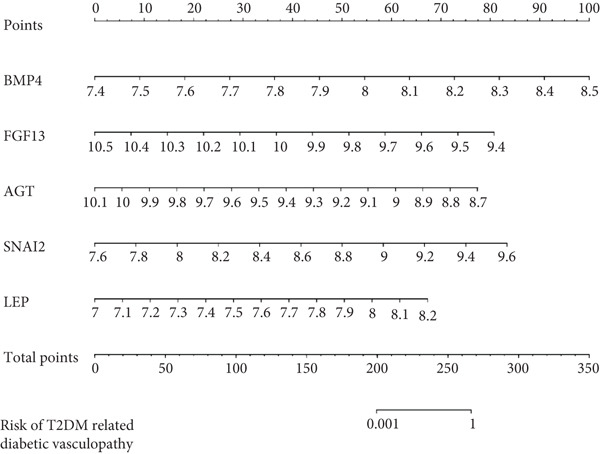
(d)

**Figure:**
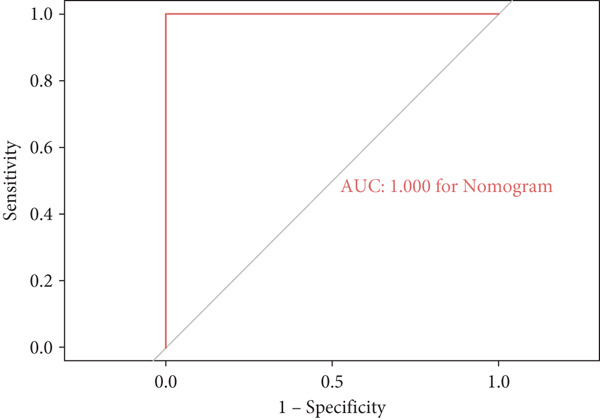
(e)

**Figure:**
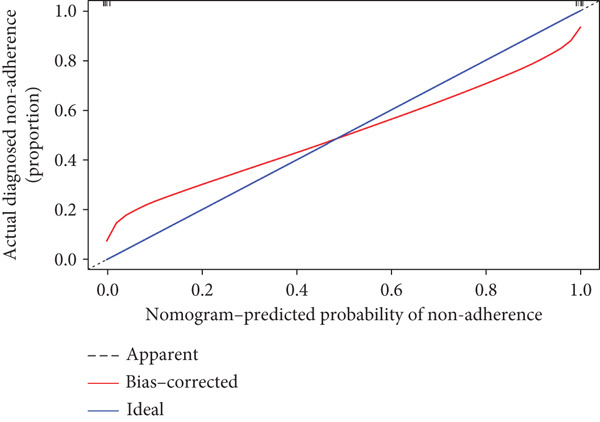
(f)

**Figure:**
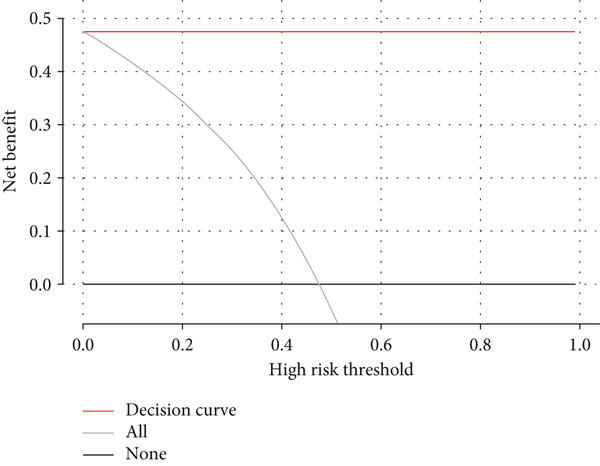
(g)

**Figure:**
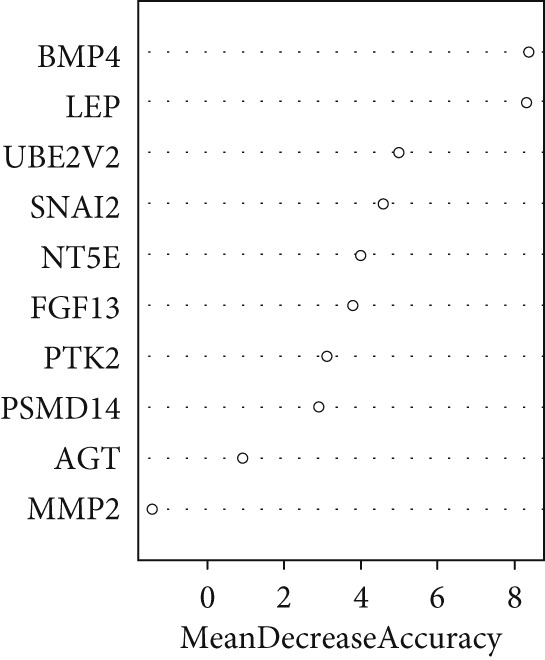
(h)

**Figure:**
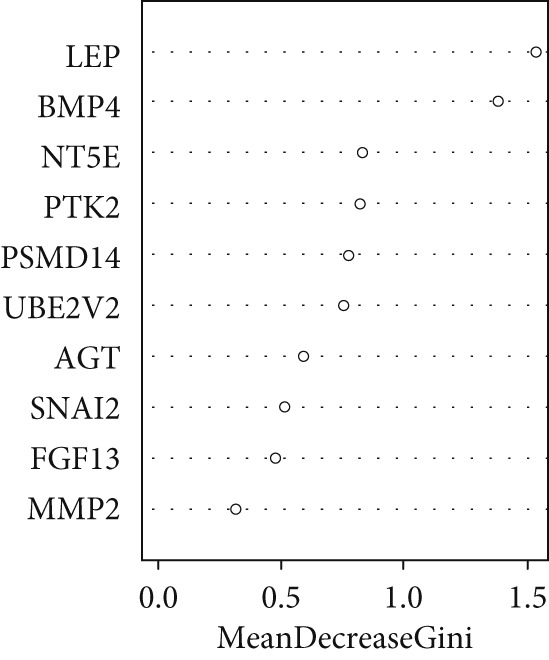
(i)

**Figure:**
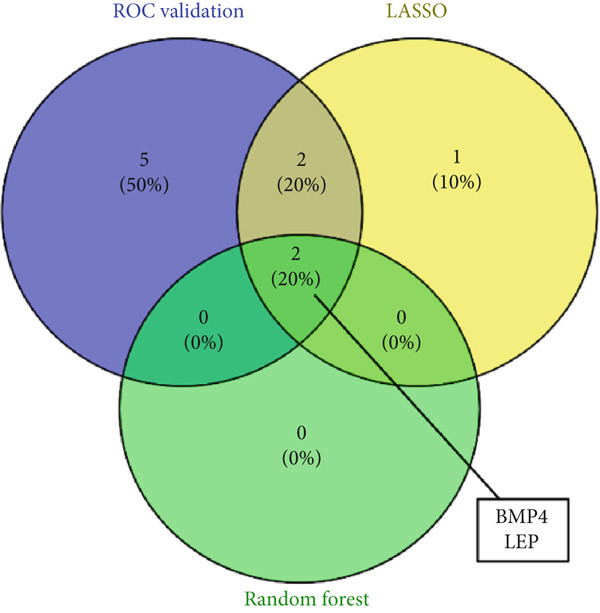
(j)

### 3.7. Construction of mRNA–miRNA, mRNA–TF Network, and JASPAR Analysis

In mRNA–miRNA network, 17 miRNAs were predicted to be targeted BMP4, and 77 miRNAs were predictably targeted LEP (Figure 10a). Interestingly, two miRNAs (has‐miR‐147a and has‐miR‐4267) were targeted both BMP4 and LEP, suggesting that these two miRNAs might play an important role on the regulation of the BMP4 and LEP expression. In mRNA–TF network, 38 TFs and four TFs, respectively, were predicted to target the genes of BMP4 and LEP (Figure 10b). Similarly, two TFs (POU5F1 and TP63) jointly targeted the expression of BMP4 and LEP, respectively. Importantly, the expression analysis for targeted TF suggested that only two TFs (TAL1 and CTCF) targeted BMP4 gene showed a statistical difference between Con and T2DM group (Figure 10c and Supporting Information 6: Table S5). Moreover, JASPAR analysis suggested that BMP4 gene carried three putative TAL1‐binding sites and two putative CTCF‐binding sites, respectively, along its DNA transcriptional regulatory region (Table 1 and Supporting Information 7: Text S1).

Figure 10Construction of mRNA–miRNA and mRNA–TF network. (a) Construction of mRNA–miRNA network for BMP4 and LEP. (b) Construction of mRNA–TF network for BMP4 and LEP. mRNA, message RNA; miRNA, microRNA; TF, transcription factor.

**Figure:**
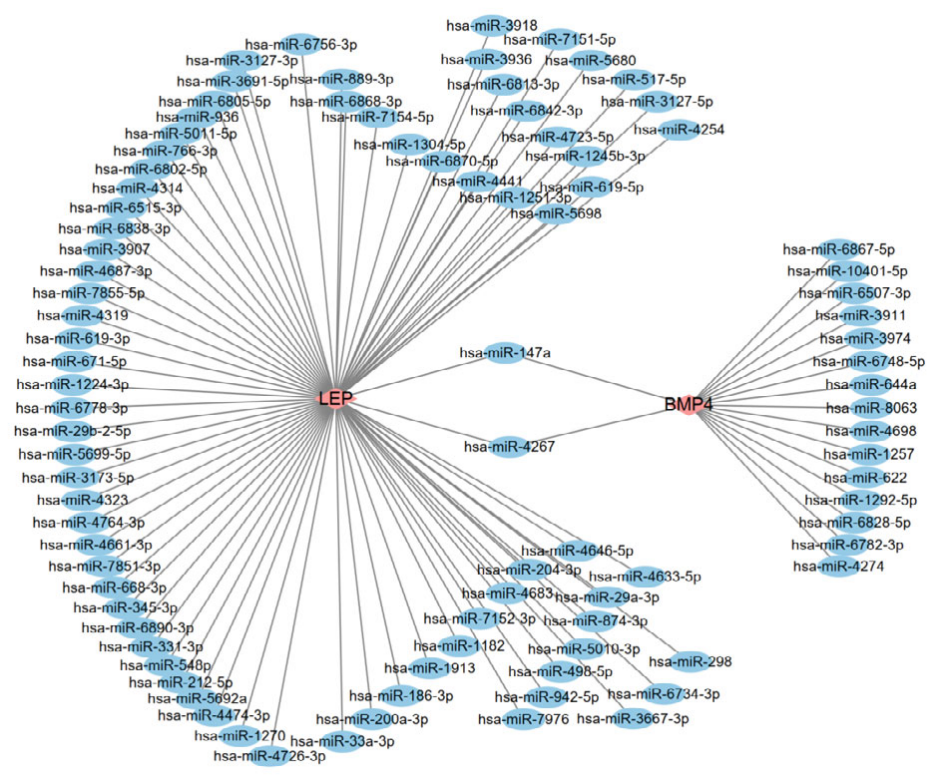
(a)

**Figure:**
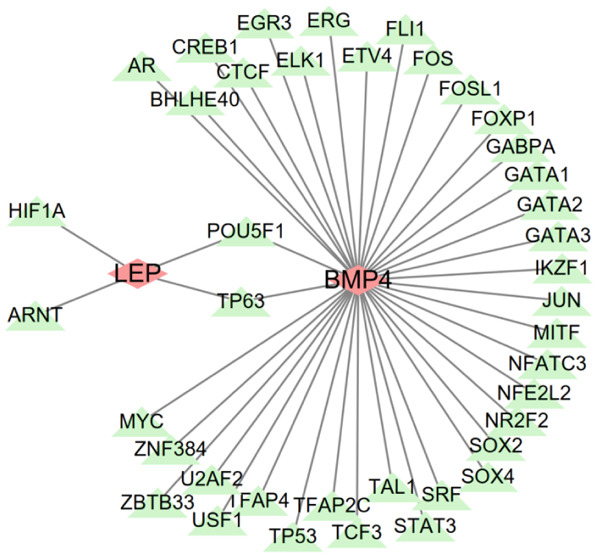
(b)

**Figure:**
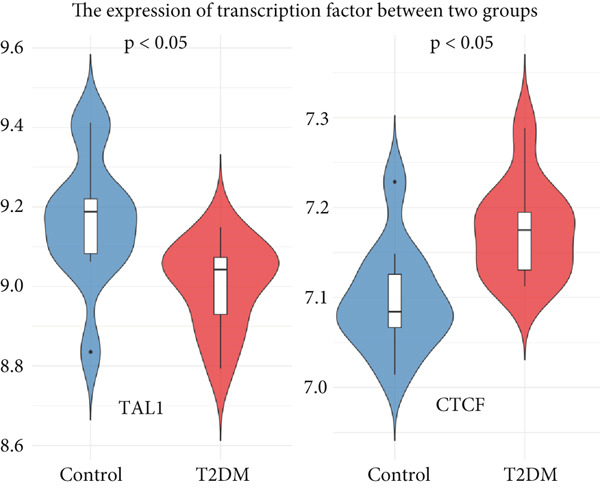
(c)

**Table 1:** JASPAR analysis results for TAL1 and CTCF binding sites located within the promoter of BMP4 gene (Sequence ID: NC_000014.9: 53947736‐53949736).

**TF**	**Relative profile score**	**Start**	**End**	**Strand**	**Predicted sequence**
TAL1	0.831	1757	1766	−	CCCATCTGGA
	0.830	1828	1837	+	ACCATCTCTC
	0.822	1886	1895	+	ACCACCTTTT

CTCF	0.819	1884	1898	−	GTCAAAAGGTGGTAC
	0.805	517	531	+	GCCAGGAGTGTGCAC

Abbreviation: TF, transcription factor.

### 3.8. Immune Infiltration Analysis

In this study, the proportion for 28 immune cell types between T2DM samples and Con samples is displayed in Figure 11a. Compared with Con samples, a higher proportion of Type 1 T helper cells and regulatory T cells was shown in T2DM samples (Figure 11b). The significant correlation between multiple immune cells was displayed with the correlation analysis of 28 immune cells, including a significant positive correlation between Type 1 T helper cells and mast cells, Type 1 T helper cells and natural killer T (NKT) cells, and regulatory T cells and effector memory CD8 T cells, while a negative correlation was displayed between regulatory T cells and immature dendritic cells, Type 1 T helper cells and immature dendritic cells, and immature dendritic cells and activated dendritic cells (Figure 11c). In addition, the BMP4 expression was positively associated with regulatory plasmacytoid dendritic cells, T cells, and Type 1 T helper cells. Meanwhile, the LEP expression was positively associated with activated dendritic cells, CD56 bright natural killer cells, and plasmacytoid dendritic cells while negatively associated with memory B cells, monocytes, and activated B cells (Figure 11d).

Figure 11Immune infiltration analysis. (a) The proportion of 28 types of immune cells between T2DM samples and Con samples. (b) The difference analysis for the proportion of 28 types of immune cells between T2DM samples and Con samples. (c) The correlation analysis between immune cells was displayed with the correlation analysis of 28 immune cells. (d) The correlation analysis of key genes (BMP4 and LEP) and infiltrated immune cells. T2DM, Type 2 diabetes milieus; Con, control.

**Figure:**
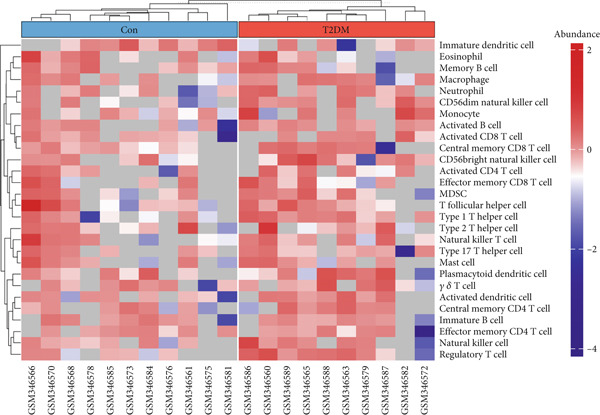
(a)

**Figure:**
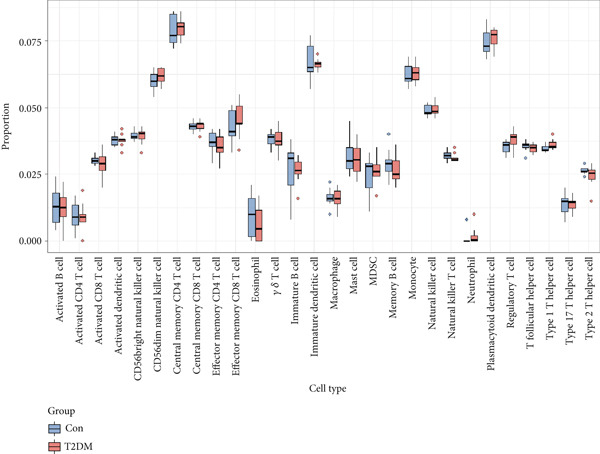
(b)

**Figure:**
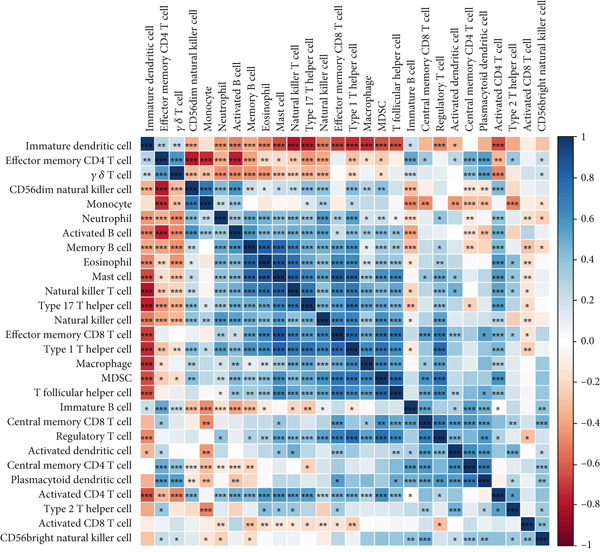
(c)

**Figure:**
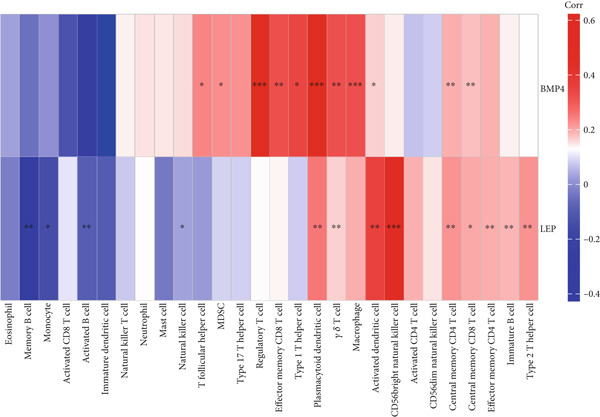
(d)

### 3.9. scRNA‐Seq Analyses Revealed BMP4 Mainly Expressed in the Endothelial Cells and Fibroblasts

To investigate the cellular composition and heterogeneity underlying diabetic vasculopathy, we analyzed scRNA‐seq data of aortic tissues from both normal and diabetic mice, utilizing two independent datasets (GSE211216 and SCP1361). In the GSE211216 dataset, UMAP analysis revealed 11 differentiated cellular subsets, including macrophages, endothelial cells, fibroblasts (Clusters 1, 2, and 3), vascular smooth muscle cells (Clusters 1 and 2), monocyte‐derived dendritic cells, T cells, B cells, monocytes, and NKT cells (Figure 12a,b). The distribution of these cell populations between normal and diabetic groups is illustrated in Figure 12c. A heatmap was generated to display the Top 5 DEGs across the major cell clusters (Figure 12d). Similar findings were observed in the SCP1361 dataset, where 10 major cell populations were identified, including dendritic cells, fibroblasts, smooth muscle cells, B cells, T cells, endothelial cells, macrophages, mesothelial cells, pericytes, and neural cells (Supporting Information 1: Figure S6A,B).

Figure 12Single‐cell RNA sequencing (scRNA‐seq) analysis revealed that BMP4 is predominantly expressed in endothelial cells and fibroblasts. (a) UMAP visualization of 11 major cell populations identified in aortic tissues from normal and diabetic mice. (b) UMAP plot displaying the distribution of cells stratified by experimental group (normal vs. diabetic). (c) Comparative UMAP analysis illustrating the differences in cell population composition between normal and diabetic mice. (d) Heatmap depicting the Top 5 differentially expressed genes (DEGs) across each cell population. (e, f) Bar graphs quantifying the absolute numbers and proportional distribution of each cell population in normal and diabetic mice. (g) Violin plot demonstrating the expression levels of BMP4 across distinct cell populations. (h) Dot plot comparing BMP4 expression levels in each cell population between normal and diabetic mice. (i) Volcano plot highlighting differentially expressed genes between BMP4high and BMP4low endothelial cells. (j) Gene Ontology (GO) functional enrichment analysis of upregulated genes in BMP4high endothelial cells. (k) Kyoto Encyclopedia of Genes and Genomes (KEGG) pathway analysis of upregulated genes in BMP4high endothelial cells.

**Figure:**
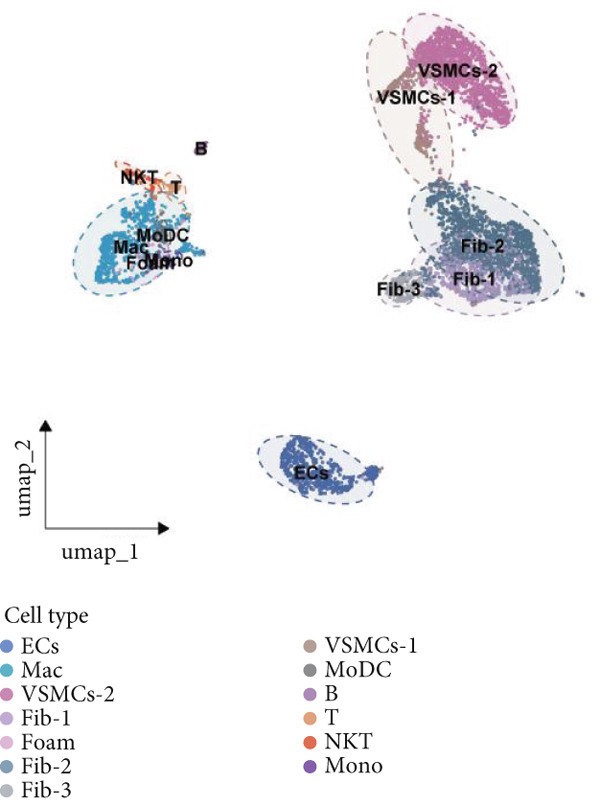
(a)

**Figure:**
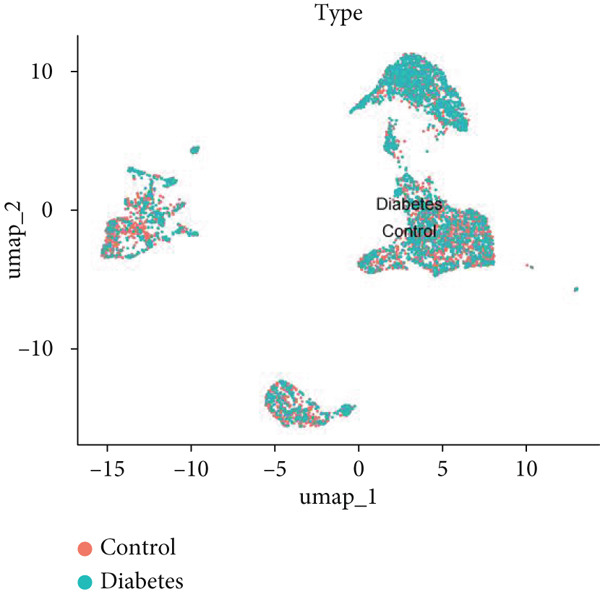
(b)

**Figure:**
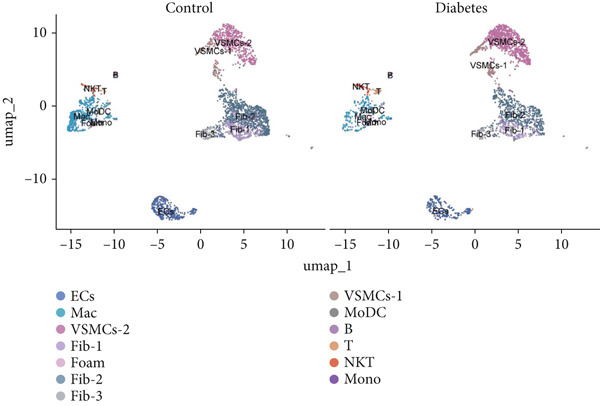
(c)

**Figure:**
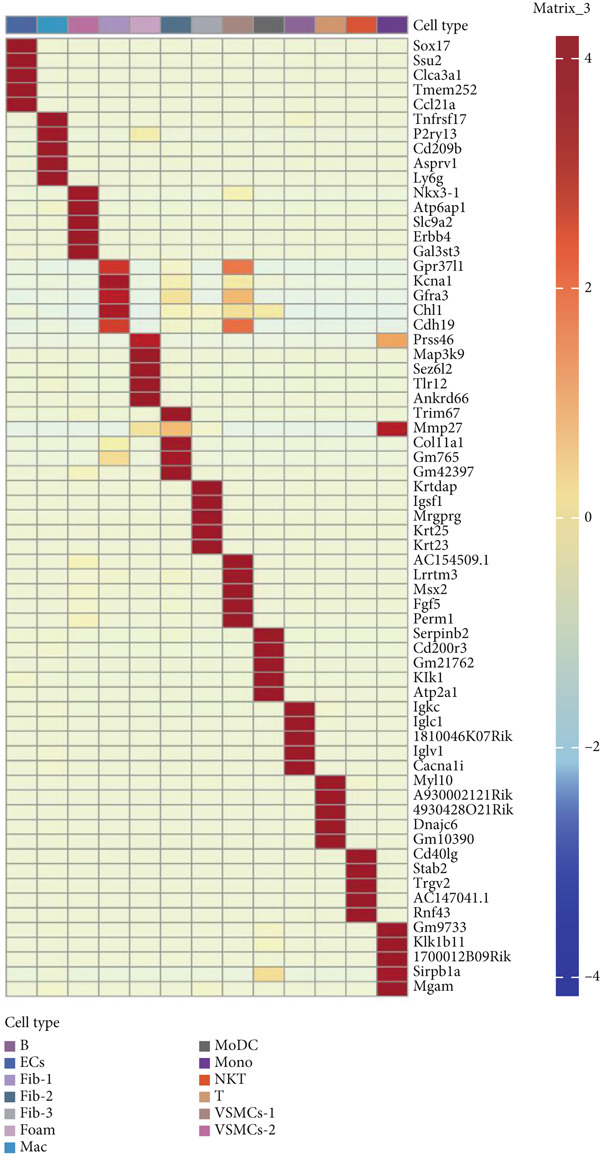
(d)

**Figure:**
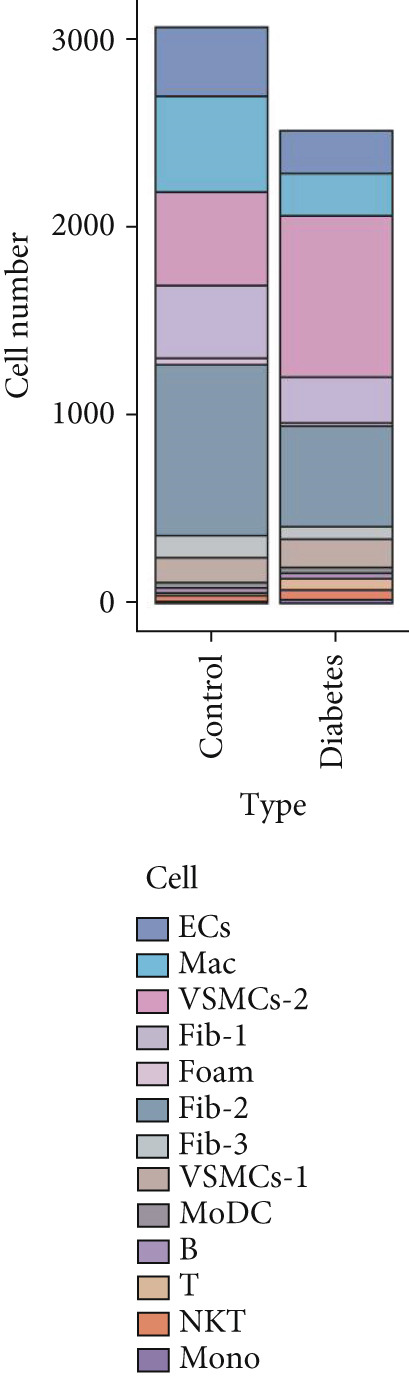
(e)

**Figure:**
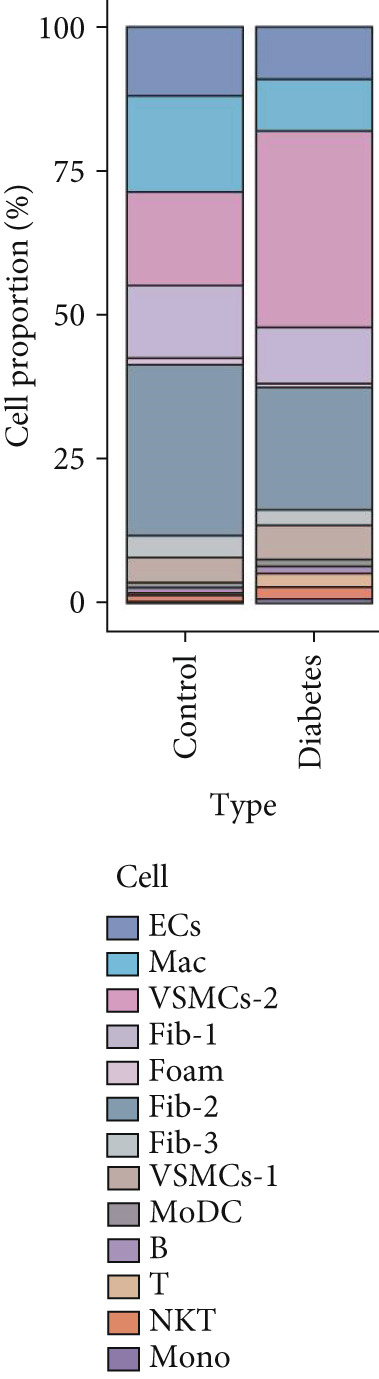
(f)

**Figure:**
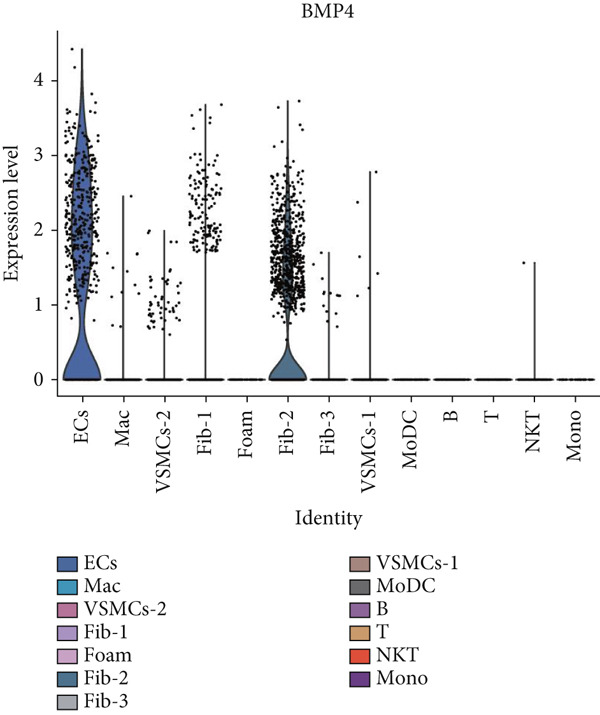
(g)

**Figure:**
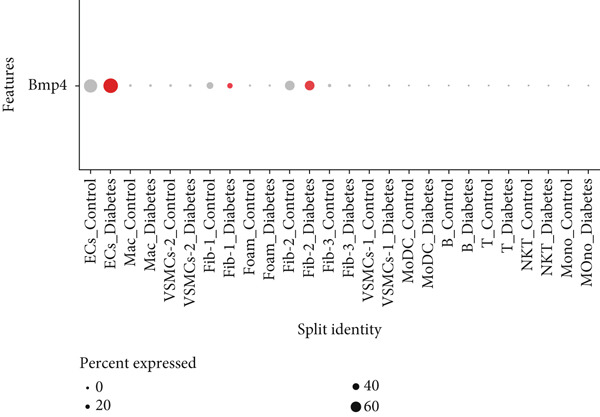
(h)

**Figure:**
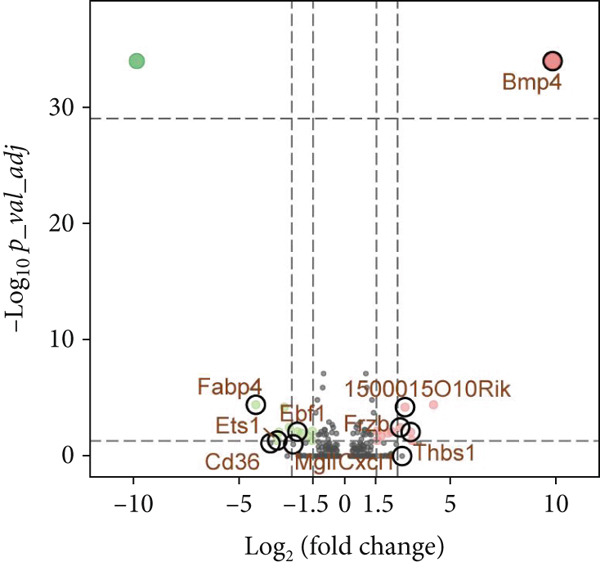
(i)

**Figure:**
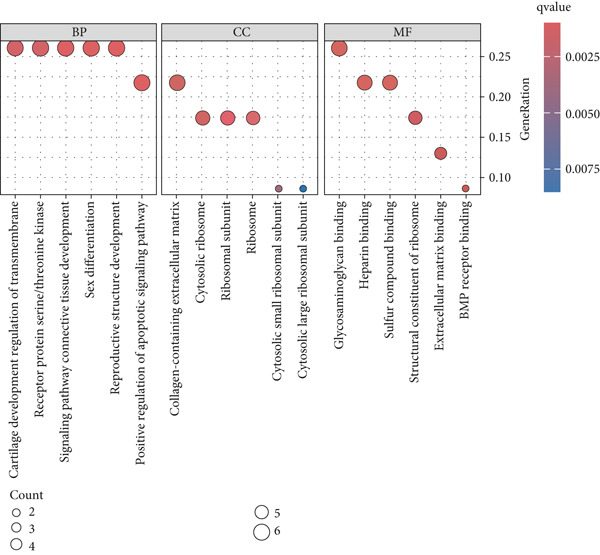
(j)

**Figure:**
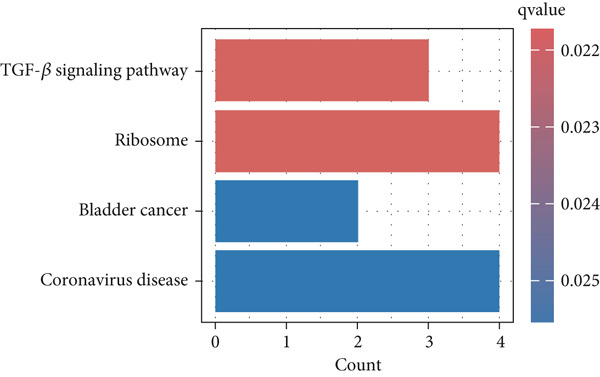
(k)

We further quantified the abundance and proportional changes of each cell population between the normal and diabetic groups (Figure 12e,f and Supporting Information 1: Figure S6C,D). Notably, vascular smooth muscle cells were significantly increased in the aortic tissues of diabetic mice compared to controls. Additionally, BMP4 expression was predominantly localized to endothelial cells and fibroblasts (Figure 12g and Supporting Information 1: Figure S6E), with significantly higher expression levels observed in the diabetic group compared to the normal group (Figure 12h and Supporting Information 1: Figure S6F).

Given the prominent expression of BMP4 in endothelial cells, we focused on elucidating the functional role of BMP4 in this cell type. Endothelial cells were stratified into two subpopulations based on BMP4 expression levels: BMP4high and BMP4low endothelial cells (BMP4high: BMP4 counts > 0; BMP4low: BMP4 counts = 0). Differential gene expression analysis between these subpopulations was performed (Figure 12i). GO and KEGG pathway enrichment analyses revealed that genes highly expressed in BMP4high endothelial cells were significantly associated with the collagen‐containing extracellular matrix, glycosaminoglycan binding, BMP4 receptor binding, and the TGF‐*β* signaling pathway (Figure 12j,k).

### 3.10. The Validation of the Key Genes of Diabetic Vasculopathy

We successfully constructed a T2DM mouse model to further validate the accuracy of the above integrated bioinformatics analysis (Supporting Information 1: Figure S5A). We found that the body weight in the T2DM group is significantly higher than that in the Con group from 2 to 4 weeks (Supporting Information 1: Figure S5B). The fast blood glucose in the T2DM group is significantly higher than that in the Con group from 6 weeks (Supporting Information 1: Figure S5C). Importantly, a significantly abnormal glucose tolerance was shown in the T2DM group at 6 weeks (Supporting Information 1: Figure S5D). The RT‐qPCR results for arterial tissue confirmed consistent upregulated expression of two key genes in diabetic arterial samples compared with the control samples (Figure 13a,b). Meanwhile, the ROC validation also showed good discrimination for the T2DM sample from the Con sample, which was consistent with the bioinformatics analysis (Figure 13c,d). According to our KEGG analysis, we focused on evaluating the fibrosis level of diabetic arteria, and the results showed that the fibrosis level of arterial tissue in the T2DM group was significantly higher than that in the Con group, which was consistent with the enrichment analysis (Figure 13e,f). Additionally, the BMP4 expression profile was further confirmed using Western blot and immunohistochemical staining. Similarly, the results suggested that compared with the Con samples, the protein expression of BMP4 was significantly increased in the T2DM samples (Figures 13g, 13h, 13i, and 13j).

Figure 13The validation of the key genes of diabetic vasculopathy. (a, b) The RT‐qPCR analysis of two key genes (BMP4 and LEP) between T2DM samples and Con samples. (c, d) The ROC validation of two key genes (BMP4 and LEP) for T2DM samples and Con samples. (e, f) The fibrosis level of arterial tissue between T2DM samples and Con samples. (g, h) Western blot analysis for BMP4 expression profile between T2DM samples and Con samples. (i, j) The immunohistochemical staining analysis for BMP4 expression profile between T2DM samples and Con samples. T2DM, Type 2 diabetes milieus; Con, control.

**Figure:**
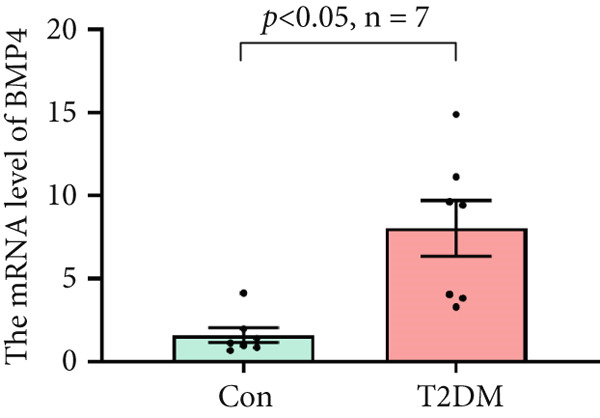
(a)

**Figure:**
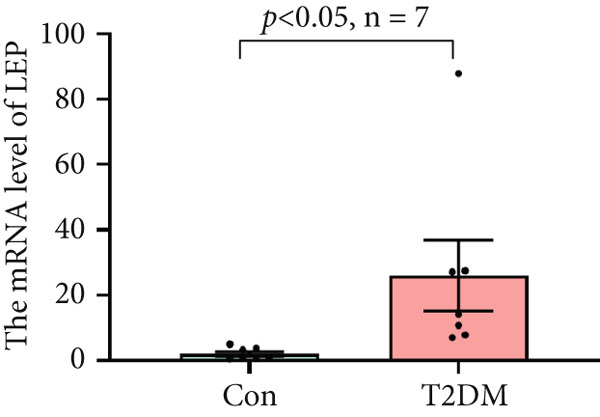
(b)

**Figure:**
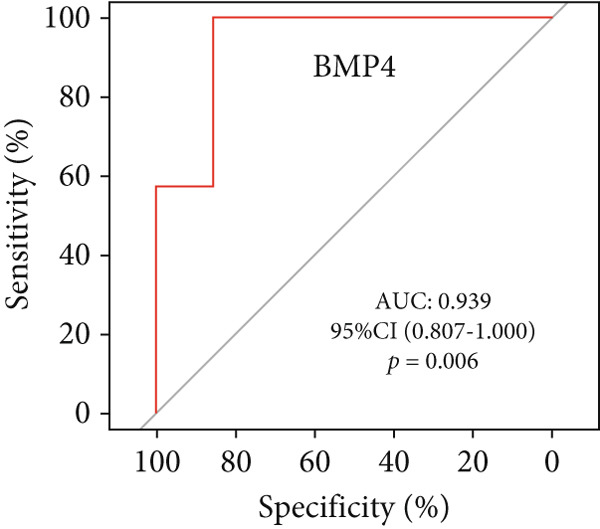
(c)

**Figure:**
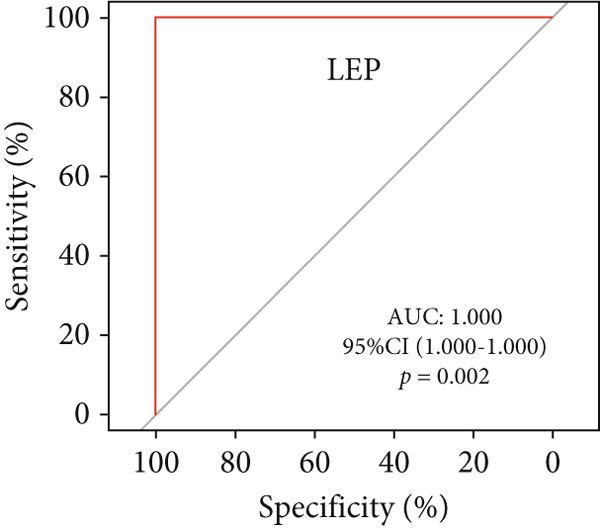
(d)

**Figure:**
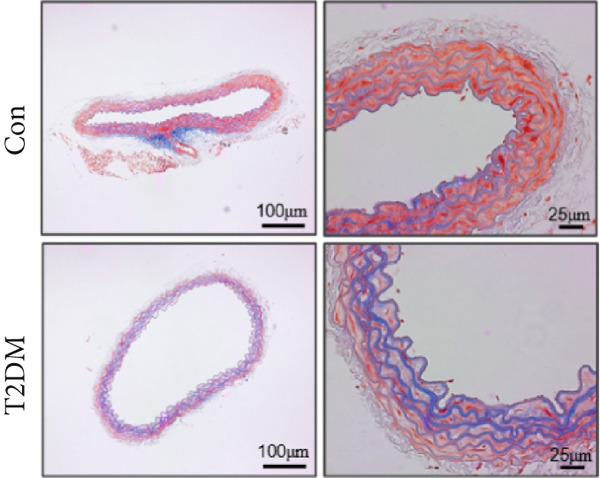
(e)

**Figure:**
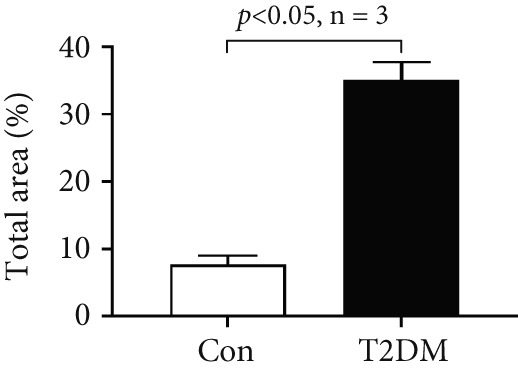
(f)

**Figure:**
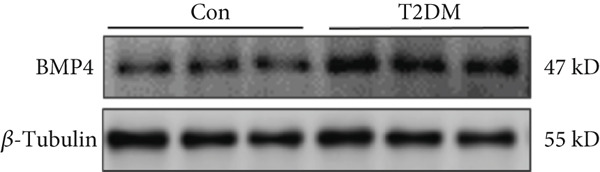
(g)

**Figure:**
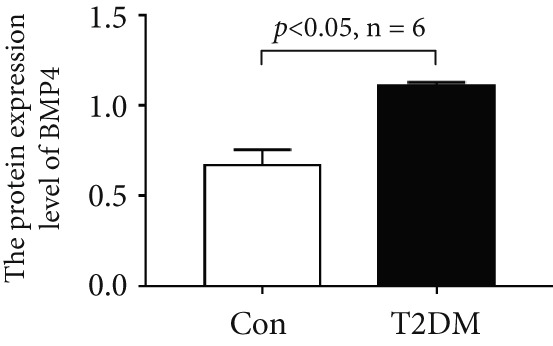
(h)

**Figure:**
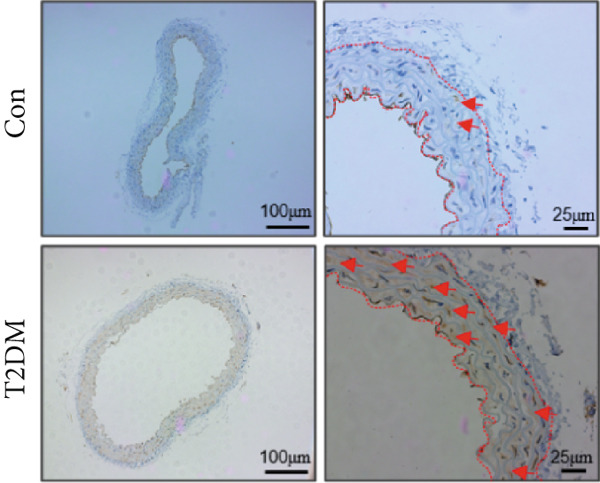
(i)

**Figure:**
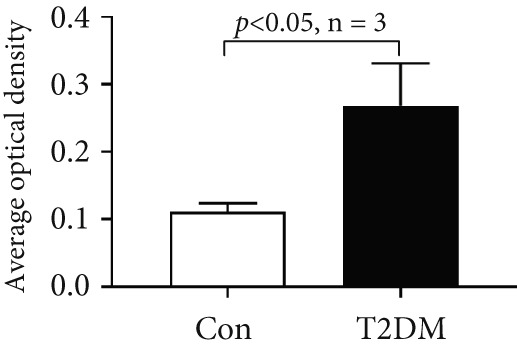
(j)

## 4. Discussion

Diabetic vasculopathy, a prevalent complication of diabetes, falls under the category of diabetic cardiovascular diseases and poses a significant risk to overall health. Utilizing the GSE13760 gene expression dataset, our analysis revealed 139 DEGs between arterial tissues from T2DM patients and control samples. We performed multiple enrichment analyses via GSEA, Metascape, and ClueGo to comprehensively explore the possible mechanisms underlying diabetic vasculopathy. Meanwhile, to identify candidate small‐molecule therapeutics for diabetic vasculopathy, we utilized the cMAP online platform with upregulated DEGs. A PPI network was subsequently established, and central hub genes were discerned by applying four distinct topological algorithms within Cytoscape. Expression profiling and coexpression relationships of these candidate hub genes were then evaluated. Crucially, core genes were definitively identified through an integrative approach combining ROC assessment, LASSO regression, and RF machine learning modeling. Furthermore, regulatory networks involving miRNAs and TFs for these core genes were elucidated, and their relationship with immune cell infiltration was explored. To empirically validate the bioinformatics predictions, an in vivo T2DM mouse model was developed and analyzed. Our main findings are as follows: (1) Two key genes (BMP4 and LEP) were screened between T2DM arterial samples and Con samples. (2) The two upregulated key genes might be involved in the pathophysiological process in diabetic vasculopathy.

Diabetic vasculopathy mainly include macrovascular disease and microvascular disease. Strikingly, macrovascular disease, a complication of T2DM involved in larger conduit arteries (including aorta, coronary, and peripheral arteries), could lead to aortic dissection, myocardial infarction, and peripheral artery disease, subsequently posing a poor prognosis to human health. Multiple potential mechanisms might contribute to the initiation and progress of diabetic vasculopathy, including immune cell activation, inflammation, oxidative stress, advanced glycation end‐products increase, and microRNAs changes.

Emerging evidence suggested that immune cell activation and inflammation underlying the T2DM progression might play an important role in the pathophysiological alternation of diabetic vasculopathy. A previous cohort study reported that the increased leukocyte level, especially neutrophils and lymphocytes, was significantly associated with insulin sensitivity and elevated C‐reactive protein in T2DM patients, thus promoting the inflammation activation of targeted tissue [18]. Elevated evidence suggested that inflammatory mediators (such as cytokines, chemokines, and adhesion molecules) could lead to cardiovascular damage due to oxidative stress, endothelial activation, and immune cell activation mediated by hyperglycemia. Hussain et al. [19] reported that hyperglycemia could induce the decreased expression of the free radical scavenger superoxide dismutase 1, as well as aldehyde dehydrogenase 2, and promote the inflammatory mediator′s expression such as NF‐*κ*B, interleukin (IL)‐1*β*, IL‐6, and TNF‐*α*, ultimately causing the lesions of vascular endothelial cells and smooth muscle cells and contributing to the development of diabetic vasculopathy. Oxidative stress, which is a key mechanism of glucotoxicity in diabetes, has been demonstrated that increased vascular reactive oxygen species (ROS) generation from multiple BPs, including activation of the renin–angiotensin system, endoplasmic reticulum stress, and mitochondrial respiration, could cause cellular damage and vascular dysfunction [20]. Moreover, elevated advanced glycation end‐products stimulate the production of ROS, NF‐*κ*B, and proinflammatory cytokines (e.g., IL‐1*β*, IL‐6, IL‐18, and TNF‐*α*). Consequently, this leads to a marked increase in intracellular ROS and triggers a synergistic oxidative stress and inflammatory cascade [21].

In this study, we performed GSEA and found that the upregulated genes in diabetic arterial samples were significantly positively associated with T2DM, predominantly suggesting that the expression of these upregulated genes was attributed to T2DM. Meanwhile, multiple inflammation‐related pathways were enriched with GSEA, such as cytokine–cytokine receptor interaction, TNF signaling pathway, and NF‐*κ*B signaling pathway, which indicated a potentially inflammatory activation in diabetic arterial samples. ECM‐receptor interaction was also enriched, and this result revealed that the process of arterial fibrosis and remodeling in diabetics might be triggered. Additionally, the upregulated genes in diabetic arterial samples might be associated with multiple metabolic disorders, including carbohydrate metabolism, nucleotide metabolism, amino acid metabolism, and ubiquitin‐mediated proteolysis.

Also, we found that the DEGs were associated with inflammation (including adaptive immune system, regulation of MAPK cascade, and neutrophil degranulation) and extracellular matrix (such as NABA ECM glycoproteins) based on the Metascape, as well as with arterial lesion‐related diseases (such as ascending aorta dilatation, postmyocardial infarction, prehypertension, and idiopathic pulmonary arterial hypertension) according to the DisGeNET database. Moreover, the enrichment analysis for DEGs based on ClueGo also showed similar results, such as the regulation of phosphatidylinositol 3‐kinase activity in BPs, as well as TGF‐*β* signaling pathway and leukocyte transendothelial migration in KEGG, suggesting that fibrosis and inflammation might play an essential role in diabetic vasculopathy. Consistent with the enrichment analysis, immune infiltration analysis showed higher immune infiltration (such as Type 1 T helper cell and regulatory T cell) in T2DM samples, suggesting that inflammation might be activated in diabetic vasculopathy. Interestingly, multiple small‐molecular drugs were screened, including PT‐630, which served as a fibroblast activation protein inhibitor; deferiprone, which served as a reducing agent; and flupirtine, which served as an apoptosis inhibitor. This result indicated that the potential small‐molecular drugs were expected to alleviate fibrosis and inflammation.

As constituent members of the TGF‐*β* superfamily, bone morphogenetic proteins (BMPs) comprise a group of more than 30 secreted ligands that interact with specific receptors, coreceptors, and signaling modulators [22]. Accumulated studies suggested that BMPs, as well as TGF‐*β* ligands, were accountable for the homeostasis and pathophysiology in the cardiovascular system. Liu et al. [23] revealed that the increased BMP4‐ROS cycle could trigger the activation of p38 MAPK/JNK/caspase 3, ultimately leading to endothelial dysfunction. Yang et al. [24] systematically reviewed the vascular calcification mechanisms regulated by BMP signaling, and osteogenic ligands BMP4 seemed to be both a marker and driver of vascular calcification. Recently, Perez‐Shibayama et al. [25] suggested that the BMP4–gremlin axis represents a potential therapeutic target for modulating myocardial inflammation, thereby reducing the risk of severe complications such as cardiac fibrosis and heart failure. However, little evidence reports the role of BMP4 in diabetic vasculopathy. In this study, we found that the mRNA and protein expression levels of BMP, as well as the fibrosis level, were significantly increased in diabetic arterial samples, suggesting that BMP4 might play an important role in aortic fibrosis induced by diabetes. Immune infiltration analysis revealed that BMP4 was positively related to multiple immune cells, indicating that immune infiltration might be associated with the aortic fibrosis induced by BMP4 elevation.

Moreover, we performed the mRNA–miRNA, as well as mRNA–TF network, and found that multiple miRNAs or TFs might modulate the BMP4 expression. Importantly, two TFs (e.g., downregulated TAL1 and upregulated CTCF) targeted BMP4 gene, which has been demonstrated with the JASPAR database, providing a potential foundation for future research on the specific mechanism of BMP4 on diabetic vasculopathy. scRNA‐seq results suggested that BMP4 is mainly expressed in the endothelial cells and fibroblasts, with the prominent level of expression observed in endothelial cells, revealing that upregulated BMP in endothelial cells played an essential role in the progression of diabetic vasculopathy.

LEP, a protein containing 167 amino acids produced by adipocytes, has been reported to be related to coronary heart disease, high body mass index, obesity, and heart failure [26]. Prolonged exposure to elevated leptin concentrations has been shown to suppress fatty acid oxidation and promote cellular uptake, thereby inducing lipid accumulation in cardiomyocytes and subsequently activating programmed cell death pathways [27]. Furthermore, LEP has been implicated in directly promoting cardiomyocyte hypertrophy and facilitating obesity‐related hypertension through sympathetic nervous system activation [28, 29]. LEP may also stimulate oxidative stress and inflammation, leading to arterial stiffness, angiogenesis, and atherogenesis [30]. In our study, we found that the LEP gene expression profile was similarly increased between diabetic arterial tissue and the GEO database, suggesting that LEP might be a key gene in diabetic arterial disease.

Several limitations should be highlighted in our study. A significant limitation is that all findings, including key gene expression and gene function analysis, are based on the microarray analysis of GSE13760, lacking validation from external databases or self‐sequencing data. This study is mainly a descriptive analysis, lacking exploration and validation of molecular mechanisms, and further experiments are needed to verify our results and demonstrate the specific mechanism of key genes on diabetic vasculopathy. We successfully constructed a T2DM mouse model and focused on the validation of BMP4 expression and the fibrosis phenotype of arterial tissue in the T2DM group. However, additional studies are warranted to illustrate the possible mechanisms between elevated BMP4 and fibrosis of diabetic arterial tissue. In addition, the eligible diabetic mouse model for scRNA‐seq analysis was induced with different approaches (STZ in GSE211216 and high‐fat diet in SCP1361). However, the scRNA‐seq analysis showed similar results between GSE211216 and SCP1361, suggesting that our results were robust.

## 5. Conclusion

We discovered elevated expression of two key genes, BMP4 and LEP, in the arterial tissues of T2DM patients relative to controls, suggesting their involvement in diabetic vasculopathy development.

## Ethics Statement

The authors have nothing to report.

## Conflicts of Interest

The authors declare no conflicts of interest.

## Author Contributions

Feng Li, Chi Geng, and Xing Xu contributed equally to this work.

## Funding

This study was funded by the National Natural Science Foundation of China (82300438), the Natural Science Foundation of the Jiangsu Higher Education Institutions of China (24KJD320003), the Discipline Construction Support Project (Phase II) for High‐Level Disciplines (XKTJ‐XK202411‐3 and SDFEYBS2310), and the Applied Basic Research (medical and health) Science and Technology Innovation Project of Suzhou (SYW2024086).

## Supporting Information

Additional supporting information can be found online in the Supporting Information section.

## Supporting information


**Supporting Information 1** Figure S1: The bar and pie charts presented the GO analysis with biological processes for the 139 DEGs based on the ClueGO plug‐in. GO, Gene Ontology. Figure S2: The bar and pie charts presented the GO analysis with cellular components for the 139 DEGs based on the ClueGO plug‐in. GO, Gene Ontology. Figure S3: The bar and pie charts presented the GO analysis with molecular functions for the 139 DEGs based on the ClueGO plug‐in. GO, Gene Ontology. Figure S4: The bar and pie charts presented the KEGG analysis for the 139 DEGs based on the ClueGO plug‐in. KEGG, Kyoto Encyclopedia of Genes and Genomes. Figure S5: Construction of the T2DM mouse model. (A) Flowchart of the construction of the T2DM mouse model. (B) The body weight between T2DM and Con mouse models. (C) The fast blood glucose between T2DM and Con mouse models. (D) The blood glucose during IPGTT between T2DM and Con mouse models. T2DM, Type 2 diabetes milieus; Con, control; IPGTT, intraperitoneal glucose tolerance test. Figure S6: scRNA‐seq analysis of the SCP1361 dataset elucidated the cellular composition and heterogeneity associated with diabetic vasculopathy. (A) UMAP visualization of 11 major cell populations identified in aortic tissues from normal and high‐fat diet‐fed (HFD) mice. (B) UMAP plot displaying the distribution of cells stratified by experimental group (normal vs. HFD). (C, D) Bar graphs quantifying the absolute numbers and proportional distribution of each cell population in normal and HFD mice. (E) Violin plot illustrating the expression levels of BMP4 across distinct cell populations. (F) Dot plot comparing BMP4 expression levels in each cell population between normal and HFD mice.

**Supporting Information 2**  

**Supporting Information 3**  

**Supporting Information 4**  

**Supporting Information 5**  

**Supporting Information 6**  

**Supporting Information 7**  

## Data Availability

The datasets generated and analyzed during the current study are available in the public Gene Expression Omnibus database (https://www.ncbi.nlm.nih.gov/geo/query/acc.cgi?acc=GSE13760). The scRNA‐seq data of aortic tissue specimens from control and diabetic mice were obtained from the NCBI GEO database (http://www.ncbi.nlm.nih.gov/geo/, Accession Number: GSE211216) and the Single Cell Portal (https://singlecell.broadinstitute.org/single_cell, Study ID: SCP1361). The data that support the findings of this study are available from the corresponding authors upon reasonable request.
