# Supplementary material for: Identification and Validation of the Key Genes of Diabetic Vasculopathy: Evidence Based on Bioinformatics Analysis and Animal Study
Source: Int J Genomics. 2025 Oct 24;2025:7850852. doi: 10.1155/ijog/7850852 (PMC12551595; doi:10.1155/ijog/7850852)

**Supplementary Figure 1. The Bar and pie charts presented the GO analysis with biological processes for the 139 DEGs based on the ClueGO plug-in**
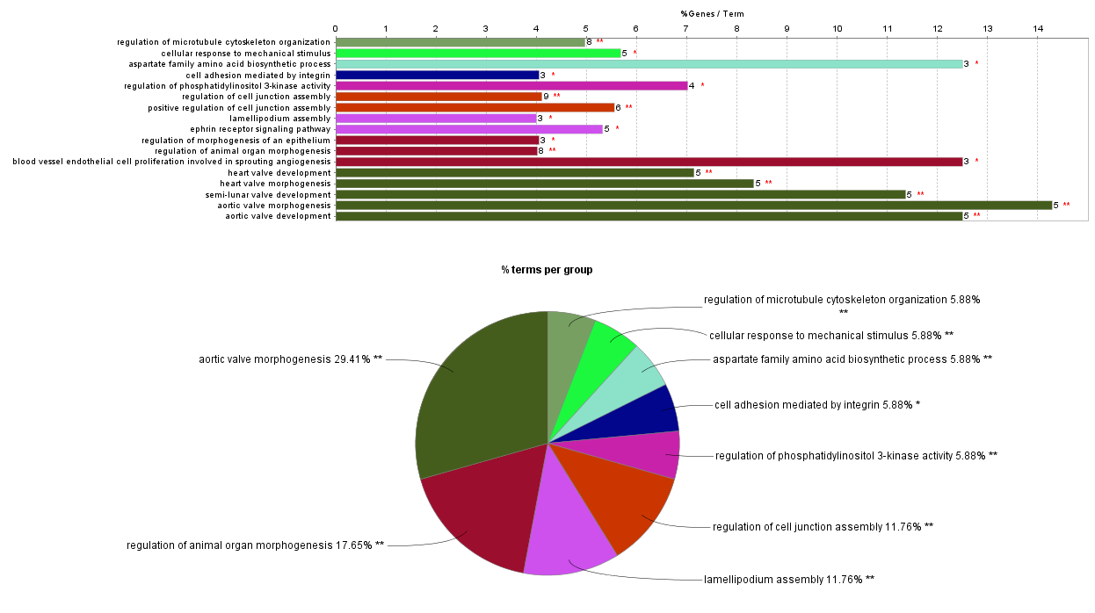


**Supplementary Figure 2. The Bar and pie charts presented the GO analysis with cellular components for the 139 DEGs based on the ClueGO plug-in**

**
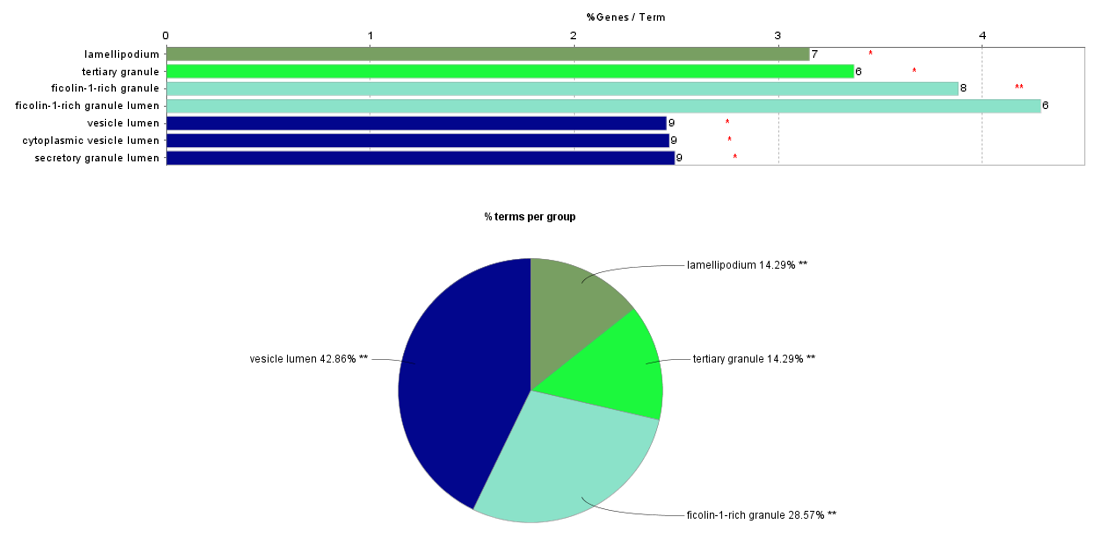
**

**Supplementary Figure 3. The Bar and pie charts presented the GO analysis with molecular functions for the 139 DEGs based on the ClueGO plug-in**

**
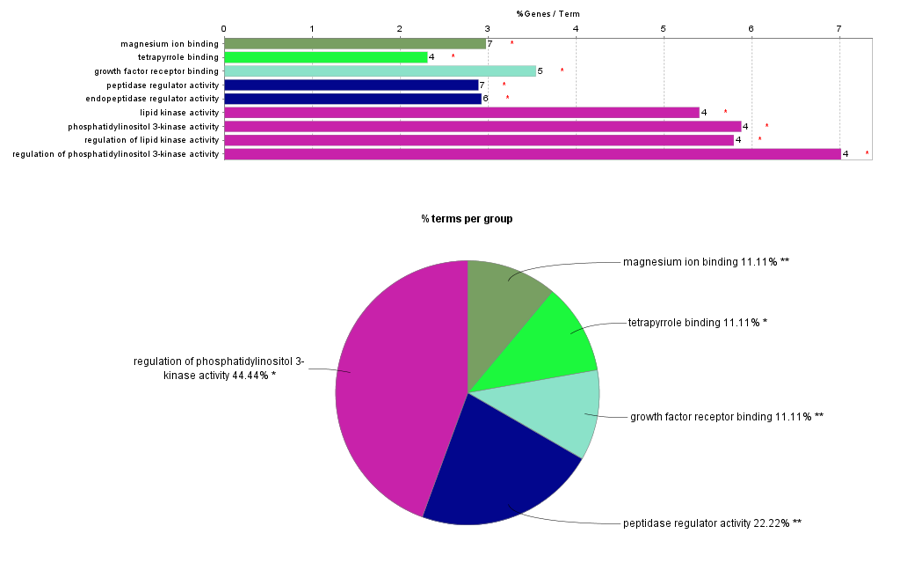
**

**Supplementary Figure 4. The Bar and pie charts presented the KEGG analysis for the 139 DEGs based on the ClueGO plug-in**

**
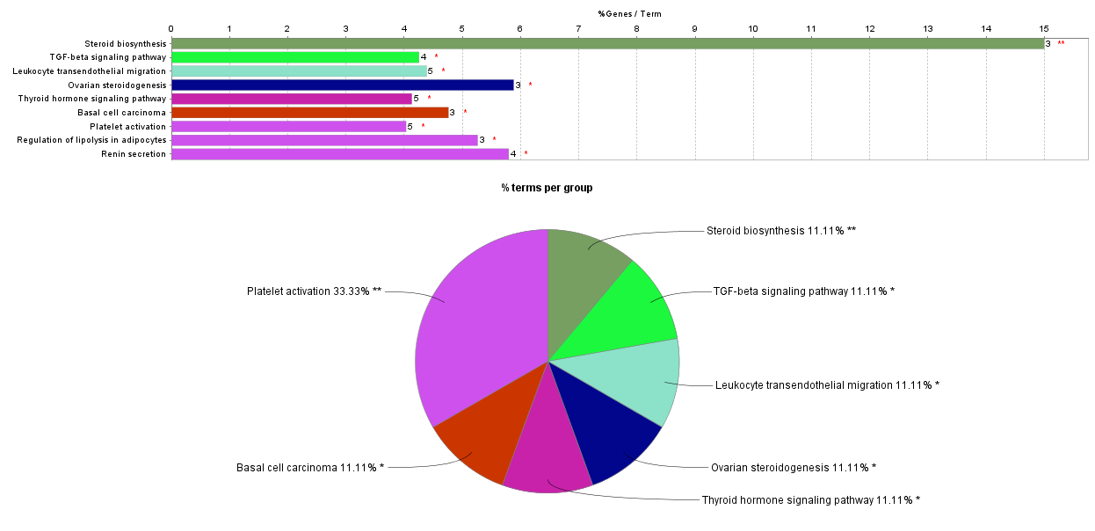
**

**Supplementary Figure 5. Construction of T2DM mice model**

**
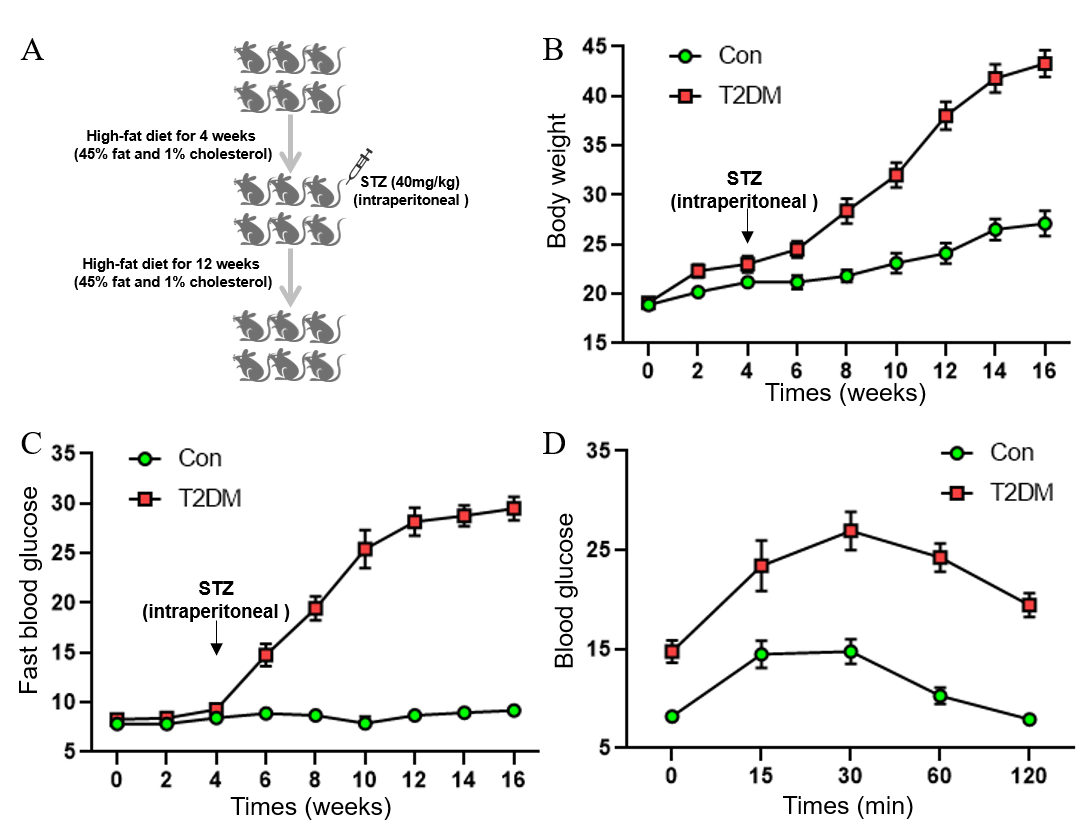
**

**Supplementary Figure 6. scRNA-seq analysis of the SCP1361 dataset elucidated the cellular composition and heterogeneity associated with diabetic vasculopathy**
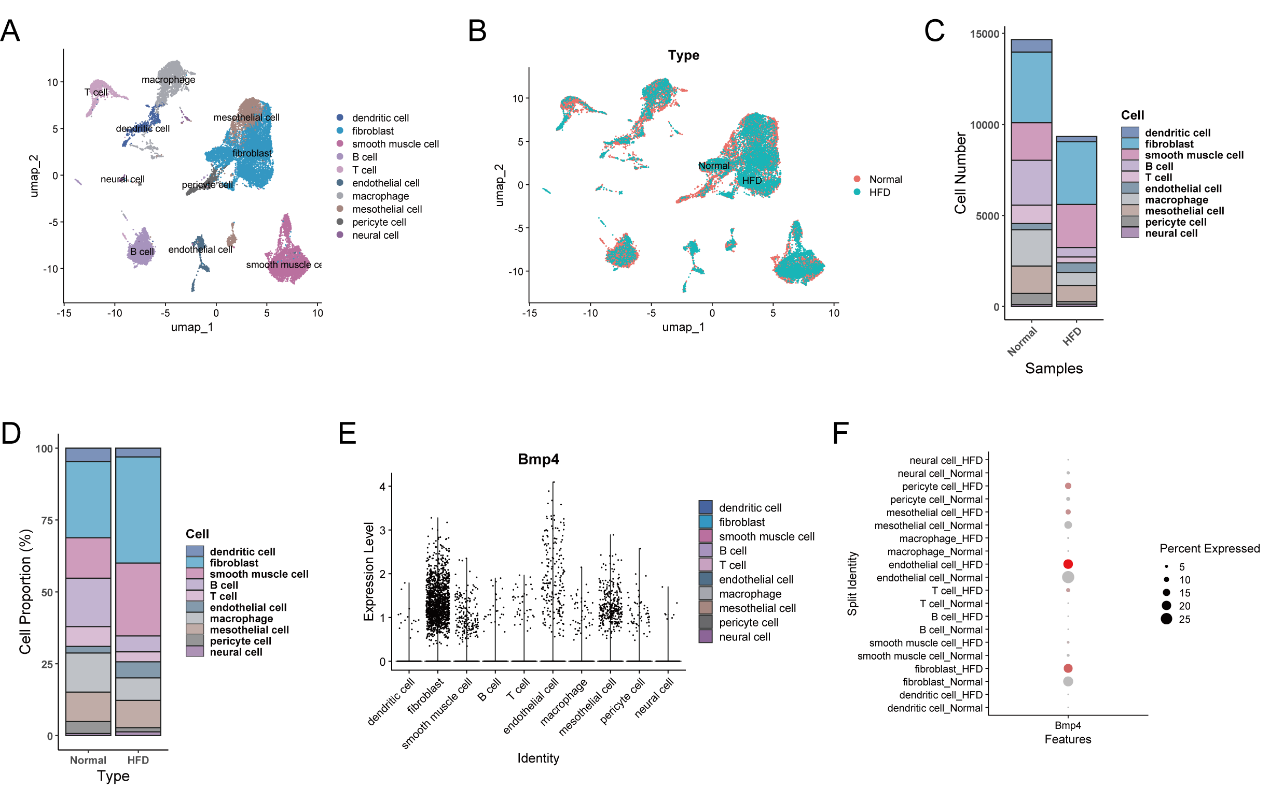

Supplement: Supplementary file 1 — Supporting Information 1 Figure S1: The bar and pie charts presented the GO analysis with biological processes for the 139 DEGs based on the ClueGO plug‐in. GO, Gene Ontology. Figure S2: The bar and pie charts presented the GO analysis with cellular components for the 139 DEGs based on the ClueGO plug‐in. GO, Gene Ontology. Figure S3: The bar and pie charts presented the GO analysis with molecular functions for the 139 DEGs based on the ClueGO plug‐in. GO, Gene Ontology. Figure S4: The bar and pie charts presented the KEGG analysis for the 139 DEGs based on the ClueGO plug‐in. KEGG, Kyoto Encyclopedia of Genes and Genomes. Figure S5: Construction of the T2DM mouse model. (A) Flowchart of the construction of the T2DM mouse model. (B) The body weight between T2DM and Con mouse models. (C) The fast blood glucose between T2DM and Con mouse models. (D) The blood glucose during IPGTT between T2DM and Con mouse models. T2DM, Type 2 diabetes milieus; Con, control; IPGTT, intraperitoneal glucose tolerance test. Figure S6: scRNA‐seq analysis of the SCP1361 dataset elucidated the cellular composition and heterogeneity associated with diabetic vasculopathy. (A) UMAP visualization of 11 major cell populations identified in aortic tissues from normal and high‐fat diet‐fed (HFD) mice. (B) UMAP plot displaying the distribution of cells stratified by experimental group (normal vs. HFD). (C, D) Bar graphs quantifying the absolute numbers and proportional distribution of each cell population in normal and HFD mice. (E) Violin plot illustrating the expression levels of BMP4 across distinct cell populations. (F) Dot plot comparing BMP4 expression levels in each cell population between normal and HFD mice. [file IJOG-2025-7850852-s003.docx]
